# Dual RNA sequencing reveals dendritic cell reprogramming in response to typhoidal *Salmonella* invasion

**DOI:** 10.1038/s42003-022-03038-z

**Published:** 2022-02-04

**Authors:** Anna Aulicino, Agne Antanaviciute, Joe Frost, Ana Sousa Geros, Esther Mellado, Moustafa Attar, Marta Jagielowicz, Philip Hublitz, Julia Sinz, Lorena Preciado-Llanes, Giorgio Napolitani, Rory Bowden, Hashem Koohy, Hal Drakesmith, Alison Simmons

**Affiliations:** 1 MRC Human Immunology Unit, MRC Weatherall Institute of Molecular Medicine, University of Oxford, Oxford, OX3 9DS UK; 2 Translational Gastroenterology Unit, John Radcliffe Hospital, Headington, Oxford OX3 9DU UK; 3 MRC WIMM Centre for Computational Biology, Weatherall Institute of Molecular Medicine, University of Oxford, Oxford, OX3 9DS UK; 4 Wellcome Centre for Human Genetics, University of Oxford, Roosevelt Drive, Headington, Oxford OX3 7BN UK; 5 Kennedy Institute of Rheumatology, University of Oxford, Roosevelt Drive, Headington, Oxford OX3 7FY UK; 6 MRC Weatherall Institute of Molecular Medicine, Genome Engineering Facility, University of Oxford, Oxford, OX3 9DS UK

**Keywords:** Pathogens, Bacterial infection

## Abstract

*Salmonella enterica* represent a major disease burden worldwide. *S. enterica* serovar Typhi (*S*. Typhi) is responsible for potentially life-threatening Typhoid fever affecting 10.9 million people annually. While non-typhoidal *Salmonella* (NTS) serovars usually trigger self-limiting diarrhoea, invasive NTS bacteraemia is a growing public health challenge. Dendritic cells (DCs) are key professional antigen presenting cells of the human immune system. The ability of pathogenic bacteria to subvert DC functions and prevent T cell recognition contributes to their survival and dissemination within the host. Here, we adapted dual RNA-sequencing to define how different *Salmonella* pathovariants remodel their gene expression in tandem with that of infected DCs. We find DCs harness iron handling pathways to defend against invading *Salmonellas*, which *S*. Typhi is able to circumvent by mounting a robust response to nitrosative stress. In parallel, we uncover the alternative strategies invasive NTS employ to impair DC functions.

## Abstract (web summary)

Aulicino, Antanaviciute et al investigate the transcriptional response to invasive Salmonella strains in dendritic cells (DCs). They show that S. Typhi mount a response against nitrosative stress pathways and propose a role of iron uptake and transport in preventing infection, which the pathogen can bypass. In parallel, they find that invasive Salmonella employs several mechanisms targeting more classic aspects of immunity to impair DC function.

## Introduction

*Salmonella enterica* serovars are responsible for a wide range of clinical presentations. *S*. Typhi is a human-restricted pathogen associated with typhoid fever, a disease that affects around 10.9 million people each year globally^1^, whilst broad host range *S*. Typhimurium is generally responsible for localised self-limiting gastroenteritis^2^. However, the multidrug-resistant *S*. Typhimurium ST313 has emerged across sub-Saharan Africa as a major cause of lethal bacteraemia in children and HIV-infected adults. ST313 pathovar D23580, a representative bloodstream clinical isolate from a Malawian child, demonstrates genome degradation resembling that of the human-restricted pathogen *S*. Typhi^3,4^, suggesting conserved genetic mechanisms by which *Salmonella* become pathogenic in humans. Nonetheless, the molecular determinants facilitating either D23580 or *S*. Typhi invasiveness remain poorly defined.

Dendritic cells (DCs) play an essential role in the initiation and establishment of antigen-specific immune responses^5^. Our group and others^6–9^, have reported that *Salmonella* can modulate DC functions to perpetuate its intracellular survival and to circumvent adaptive immunity.

Dual RNA sequencing (RNA seq) enables simultaneous gene expression profiling of infecting bacteria and infected host cells, capable of defining bacterial and host cross-talk^10–15^. However, due to the small relative fraction of bacterial mRNA, current methods for dual host–pathogen RNA seq can fail to obtain the required coverage of pathogen transcriptomes necessary to accurately reveal the nature of these complex molecular interactions, in particular in systems where only very low numbers of bacteria are present within the host cell. Recent enrichment strategies based on hybridisation selection of bacterial targets allowed more accurate bacterial gene expression quantification^16,17^.

In this study, using a custom hybridisation probe design, we enriched for *Salmonella* mRNA transcripts from infected human DCs. In contrast to previous methods, we designed our target probes to capture both conserved and divergent regions of three *Salmonella* strains in a single panel, enabling enrichment directly from pooled libraries in a single reaction. By doing so, we increased bacterial transcriptome coverage and depleted unwanted, highly abundant RNA species. Our method showed improved accuracy of bacterial gene expression quantification while also enabling the discovery of novel aspects of host–pathogen interactions and more robust comparisons due to minimised biases between strains.

Additionally to its importance in host cell function, iron regulation is also a crucial host defence weapon, where it is employed as a strategy to limit iron availability to pathogens (known as nutritional immunity^18^) or to trigger innate immune defence mechanisms, such as the production of reactive oxygen and nitrogen intermediates (ROI, RNI)^19^.

The interplay between macrophage nutritional immunity and intracellular bacterial iron acquisition has been extensively studied^18,20,21^. In contrast, little is known about the importance of iron for dendritic cell function during bacterial infection and how different *Salmonella* strains might manipulate these pathways in dendritic cells to avoid immunity.

In this study, we clarify the molecular mechanisms of virulence imparted by invasive *Salmonella* strains, particularly highlighting iron’s importance in their interaction with human DCs.

We show that *S*. Typhi is able to mount a robust response against nitrosative stress pathways induced in DCs. We propose a model where DC iron acquisition and appropriate intracellular trafficking are important host weapons to prevent intracellular bacterial infection, which the human pathogen *S*. Typhi is able to bypass. In parallel, we provide evidence that invasive non-typhoidal *Salmonella* employs alternative strategies of immunomodulation to impair DC functionality.

In summary, we provide the first analysis of the transcriptional response of invasive *Salmonella* during infection of human DCs. Our methodology has widespread utility in defining host–pathogen cross-talk more generally and identifying novel therapeutic targets for infections with limited treatment options.

## Results

### Dual RNA sequencing of *Salmonella*-infected MoDCs

**Fig. 1:**
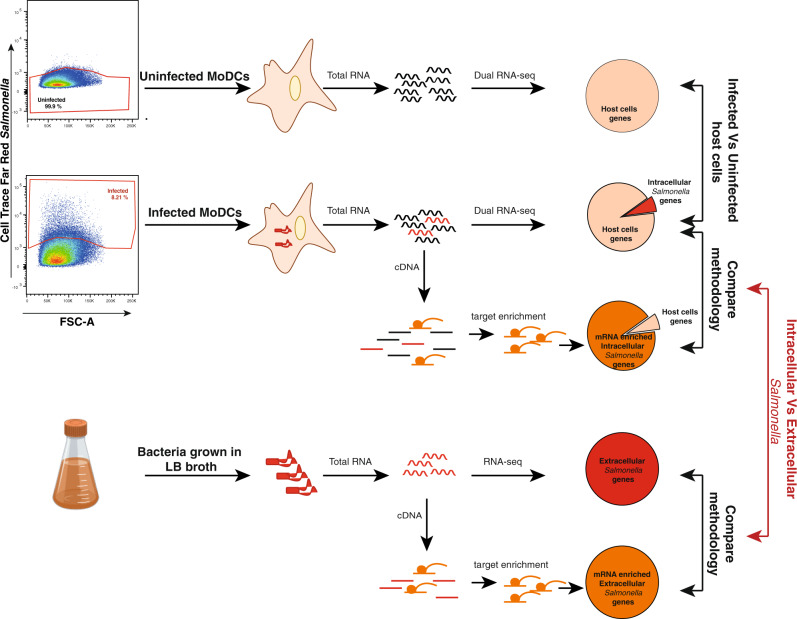
Dual RNA seq of human MoDCs challenged with invasive or non-invasive *Salmonella*. Schematic representation of the experimental design. Human MoDCs were challenged with labelled bacteria. At 6 h post infection, infected cells or uninfected cells were sorted and total RNA was extracted. Libraries were prepared according to the SMARTer stranded kit and human rRNA was depleted. Libraries were then hybridised with the *Salmonella*-specific probes or left unenriched before sequencing. Libraries prepared from RNA extracted from bacteria grown in LB broth were used as control.

To profile the transcriptional responses of both host and pathogen during infection we labelled *Salmonella* with CellTrace^TM^ Far Red Cell Proliferation dye prior to infection, as previously described^9^. MoDCs that engulfed *Salmonella* could be identified by their emitted red fluorescence, while bystander MoDCs exhibited no fluorescent signal (Supplementary Fig. 1a). We confirmed the presence of live *Salmonella* within infected cells by sorting MoDCs by their fluorescence phenotype and enumerating intracellular bacteria after cell lysis (Supplementary Fig. 1b). We used fluorescence-activated cell sorting (FACS) to isolate 20,000 infected or uninfected monocyte-derived DCs (MoDCs) at 6 h post infection (p.i.) (Supplementary Fig. 1a and Fig. 1), when the bacteria have adapted to the host intracellular environment and activated mechanisms to escape immune surveillance^22,23^.

As bacterial transcripts in infected MoDCs represent only a small fraction of total RNA within the host cell, we first devised an enrichment strategy to increase the yield of bacterial RNA reads. We designed one set of probes targeting conserved and divergent regions of STM-D23580, ST-Ty2 and STM-LT2 transcriptomes, covering all annotated genes with the exception of highly abundant tRNA and rRNA species ('Methods'). This strategy enables the depletion of both host RNAs and unwanted bacterial RNAs. By targeting conserved regions (where possible) and using a common probe set, our approach minimises potential bias that could otherwise arise from a) different batches and variation in probe composition and b) differences in capture and sequencing efficiency from more divergent regions of ortholog transcripts between different species of *Salmonella*. Our design yielded 17,570 total probes with total coverage of 5.112 Mbp across three bacterial transcriptomes (Supplementary Data 1).

Next, total RNA was isolated from both sorted infected cells and from bacteria grown in the liquid broth that was used as control (Fig. 1). Libraries were prepared according to the SMARTer^®^ stranded kit and then split and hybridised with the *Salmonella*-specific probes or left unenriched before sequencing.

All sequenced reads were aligned against a joint *Salmonella*-human genome reference and while there was some variability in individual libraries, 10% median alignments were obtained for STM-D23580, 18% for STM-LT2 and 3% for ST-Ty2 in unenriched libraries (Supplementary Fig. 2a). The majority of these constituted rRNAs and tRNAs. Conversely, following our enrichment strategy, the majority of the sequenced libraries (98–99%) were comprised of bacterial RNAs in all three strains (Supplementary Fig. 2a). To assess the sensitivity of our approach, we first downsampled all libraries to either 1 million or 10 million total reads. We quantified the total number of bacterial genes detected at minimum 10 or 50 reads per transcript thresholds, as RNA-seq data are highly heteroskedastic and thus lower coverage transcripts often exhibit too high variance for robust comparisons. We found that by investing 1 M sequencing reads per library, across different sample groups, we could detect between 57–129 median genes (10 reads minimum) in unenriched libraries and 517–869 in enriched and similar gains in enriched data were seen at higher coverage (Supplementary Fig. 2b, c), thus highlighting the gains in meaningful bacterial transcript yield and sequencing economy. Next, we compared each bacterial locus between enriched and unenriched libraries, obtained from bacteria grown in LB broth and from infected cells. In each case, the most significant differences corresponded to bacterial rRNA or tRNA transcripts, which were successfully depleted (at least 32-fold in most of these transcripts) by negative selection (Supplementary Fig. 2d–g).

We carried out principal component analysis (PCA) of all samples and found that while the second PC separated samples by enrichment strategy, the first PC, which accounted for the majority of the variability in the data in each of the three *Salmonella* strains, captured differences between bacteria grown in LB broth and intracellular bacterial transcriptomes (Supplementary Fig. 2h–j). Similarly, when we compared the results of differential expression analyses in enriched and unenriched libraries, we found the log_2_ fold changes to be highly correlated (Supplementary Fig. 2k, l). Taken together, this suggests that our enrichment strategy preserved the core biological signal of the data, while greatly improving the sequencing economy and depleting unwanted bacterial RNA species.

### Strain-independent host–pathogen response

*Salmonella* has the ability to tightly regulate its gene expression in response to environmental changes. To draw a global picture of bacterial gene regulatory processes employed during host cell infection, we compared the intracellular bacterial expression profiles from infected cells to that of *Salmonella* cultures harvested at the mid-logarithmic phase.

**Fig. 2:**
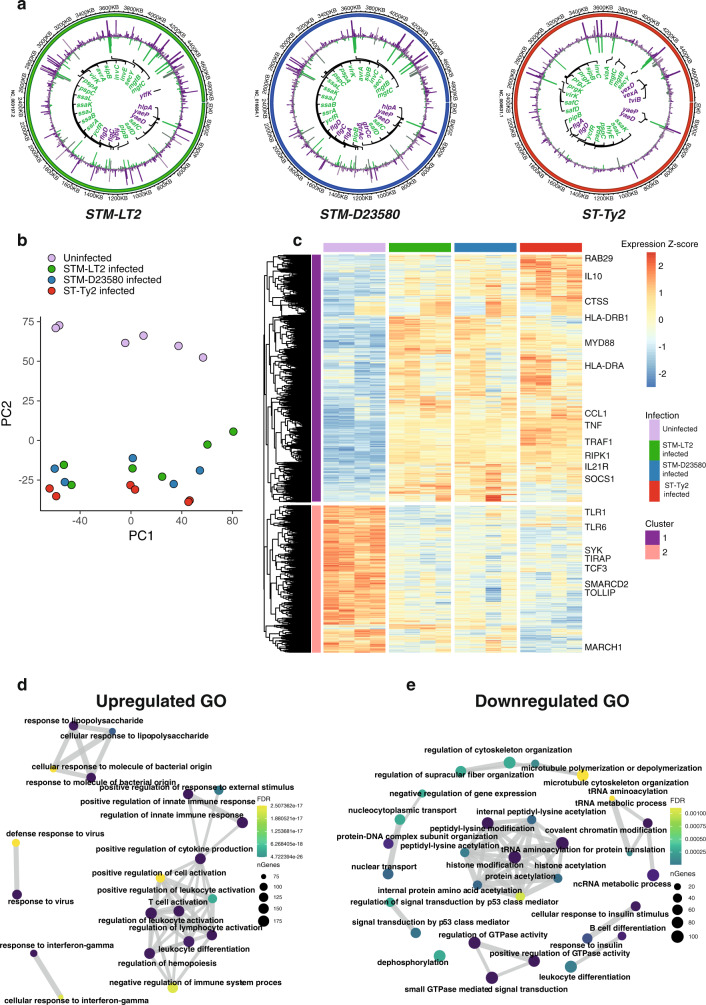
Global host–pathogen response. **a** Circos plots representing the three *Salmonella* chromosomes with genes plotted over chromosomal coordinates. Normalised expression values of RNA-seq data are displayed. Selected differentially expressed (DE) genes between extracellular (violet) and intracellular (green) conditions are indicated. Muted green and purple respectively show intracellular and extracellular expression of non-significantly (<5% FDR) differentially expressed genes. **b** Principal component analyses (PCA) of MoDCs showed a clear separation between infection-associated responses. The sorted subpopulations are colour coded. **c** Heatmap representation of all significantly differentially expressed human host genes between the three *Salmonella* strains and uninfected cells, highlighting genes that drive heterogeneity across the experimental conditions. Library-size normalised, within gene scaled, variance-stabilised counts are plotted. Row dendrogram is cut into two clusters, with cluster 1 broadly grouping all host genes upregulated in response to infection and cluster 2 showing downregulated genes. Selected genes are shown as labels. **d** Network plot of top 20 most significantly enriched Gene Ontology Biological Process terms in genes upregulated in response to *Salmonella* infection (see panel **c**, cluster 1). Node size represents the number of DE genes in category and colour indicates enrichment FDR value. **e** Network plot of top 20 most significantly enriched Gene Ontology Biological Process terms in genes downregulated in response to *Salmonella* infection (see panel **c**, cluster 2). Node size represents the number of DE genes in category and colour indicates enrichment FDR value.

Intracellular bacteria showed elevated expression of infection-associated genes, encoding for *Salmonella* Pathogenicity Island (SPI)-2 and SPI-3 effector proteins as well as the typhoidal toxin *hlyE*, but downregulated expression of genes encoding for SPI-1 factors, flagella and capsule’s biosynthesis (Fig. 2a and Supplementary Data 2). These results agree with previous large-scale transcriptional studies of *Salmonella* infection^10^.

In parallel, significant modifications of the host transcriptome were detected in infected as compared with uninfected MoDCs as confirmed by PCA (Fig. 2b and Supplementary Data 3). All *Salmonella* strains induced host genes coding for proteins involved in immune responses and characterised by antimicrobial activity (*TNF, IL1B, CCL1, CCL4, HLA-DRA, CTSS, GBP1)*. In contrast, downregulated genes in *Salmonella*-infected MoDCs were mostly involved in transcription and protein synthesis (*HIST1H1C, MTA2, SMARCD2, CIITA, CEBPA, TCF3)*, consistent with previous studies showing inhibition of host protein synthesis as a common strategy used by intracellular pathogens to disrupt host gene expression^24,25^ (Fig. 2c–e and Supplementary Data 4).

### Dendritic cells reprogramming in response to invasive *Salmonella* serovars

To identify unique differences between MoDCs infected with the three bacterial strains, we directly compared host cell gene expression between STM-D23580, STM-LT2 and ST-Ty2-infected MoDCs. We found 2265 genes (false discovery rate, FDR < 5%) differentially expressed (DE) between STM-LT2- and ST-Ty2-infected MoDCs, 1757 DE genes between STM-D23580 and ST-Ty2-infected MoDCs, but only 450 genes were found being DE between STM-LT2 and STM-D23580-infected MoDCs (Supplementary Fig. 3 and Supplementary Data 5).

**Fig. 3:**
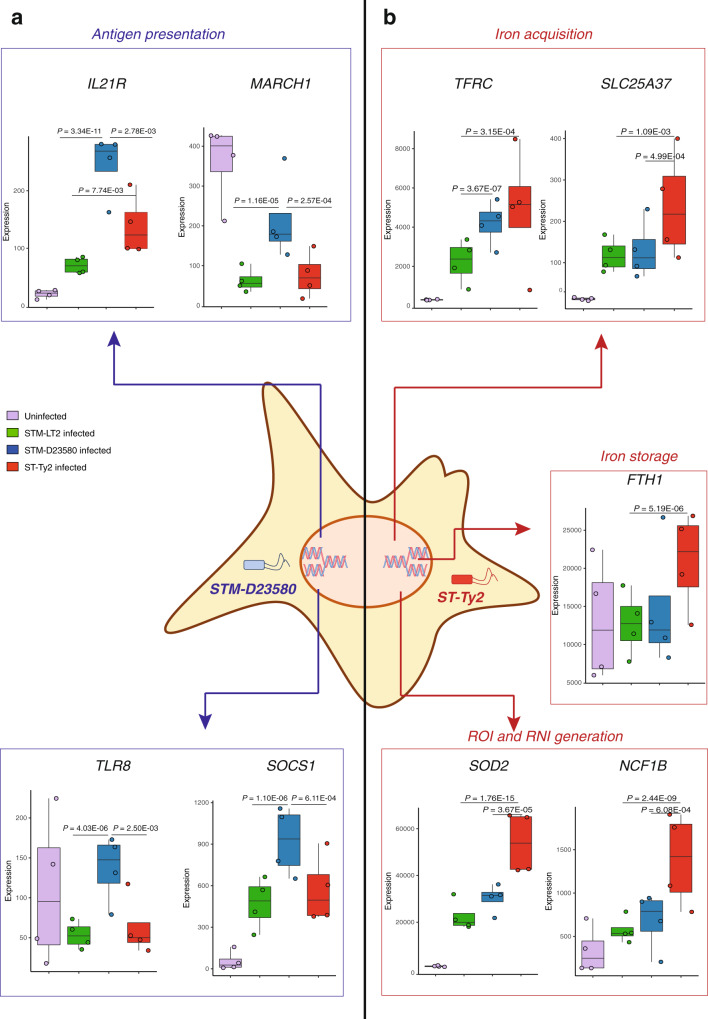
Unique host response to invasive *Salmonella*. **a** Box plots displaying the normalised gene expression level of *MARCH1, IL21R, TLR8, SOCS1* in infected and uninfected cells. **b** Box plot displaying the normalised gene expression level of relevant DE genes identified between ST-Ty2-infected cells and *S*. Typhimurium-infected or uninfected MoDCs.

The differences identified in gene expression in STM-D23580 versus STM-LT2 or ST-Ty2-infected MoDCs suggested that invasive non-typhoidal *Salmonella* can adopt specific mechanisms of immunomodulation in DCs to facilitate systemic spread. In agreement with our previous observations^9^, we found that the RING type E3-ubiquitin Transferase *MARCH1*, required for ubiquitination of MHC-II molecules, was significantly downregulated in STM-LT2 or ST-Ty2-infected cells when compared with uninfected or STM-D23580-infected MoDCs. Furthermore, STM-D23580 infected MoDCs significantly upregulated the expression of the interleukin 21 receptor (*IL21R)*, *TLR8*, and suppressor of cytokine signalling 1 (*SOCS1)*, as compared with uninfected and STM-LT2 or ST-Ty2-infected cells. (Fig. 3a and Supplementary Data 5).

A distinct set of genes uniquely modulated by ST-Ty2-infected MoDCs included: *RAB29*, a known marker of *S*. Typhi-containing vacuoles^26^, the biogenesis of lysosome-related Organelle Complex-3 (*BLOC1S3*), which may regulate *S*. Typhi replication in human macrophages^27^. In addition, *S*. Typhi-infected cells expressed higher levels of *STAT6* and *IL4R*, a marker previously associated with murine macrophages infected with growing *S*. Typhimurium^28^ and upregulated by the *Salmonella* effector protein SteE^29^ (Supplementary Data 5).

To validate some key genes identified as differentially expressed between *S*. Typhi and *S*. Typhimurium-infected MoDCs, we used a broader selection of bacterial isolates, including a multidrug-resistant strain of *S*. Typhi (ST-CT18), *S*. Typhimurium strain 4/74 (STM-4/74) and one other *S*. Typhimurium ST313 strain (STM-D37712). Our results confirmed that both the Typhoidal strains induced a higher expression of genes in the iron pathway (*SOD2* and *SLC25A3*) and the immunoregulatory cytokines *IL6* and *IL10* (Supplementary Fig. 4).

Finally, to better characterise the nature of the host response to the different *Salmonella* strains, we also inspected long non-coding RNA (lncRNA) expression changes after infection, as lncRNAs are implicated in the host response to *Salmonella* infection^10,30^. We observed an extensive rewiring of non-coding transcriptome, and immune-associated lncRNAs were strongly induced in response to *Salmonella* infection (Supplementary Fig. 5 and Supplementary Data 6).

Interestingly, among the lncRNAs that were significantly upregulated in *S*. Typhi-infected MoDCs we found *LUCAT1*, which acts downstream of NRF2 to regulate gene expression and mediate oxidative stress protection^31^, and *LINC01137* that has been reported to increase after exposure to hydrogen peroxide (H_2_O_2_)^32^.

### Strain-dependent remodelling of cellular iron homoeostasis

Macrophage iron handing is critical for the control of intracellular infections^33,34^. However, it remains unclear whether iron metabolism constitutes an innate defence mechanism in DCs. Interestingly, our dual-RNA seq highlighted how iron metabolism was differentially regulated between DCs infected with different stains.

*S*. Typhi-infected cells overexpressed genes associated with iron uptake (*TFRC, STEAP3, SLC25A37*) and storage (*FTH1*). Also, MoDCs infected with ST-Ty2 showed a marked downregulation of antioxidant genes (*TXNIP*, *TXNRD1*) and induction of genes involved in ROI and RNI formation (*NCF1B, SOD2*) (Fig. 3b and Supplementary Data 3 and 5).

**Fig. 4:**
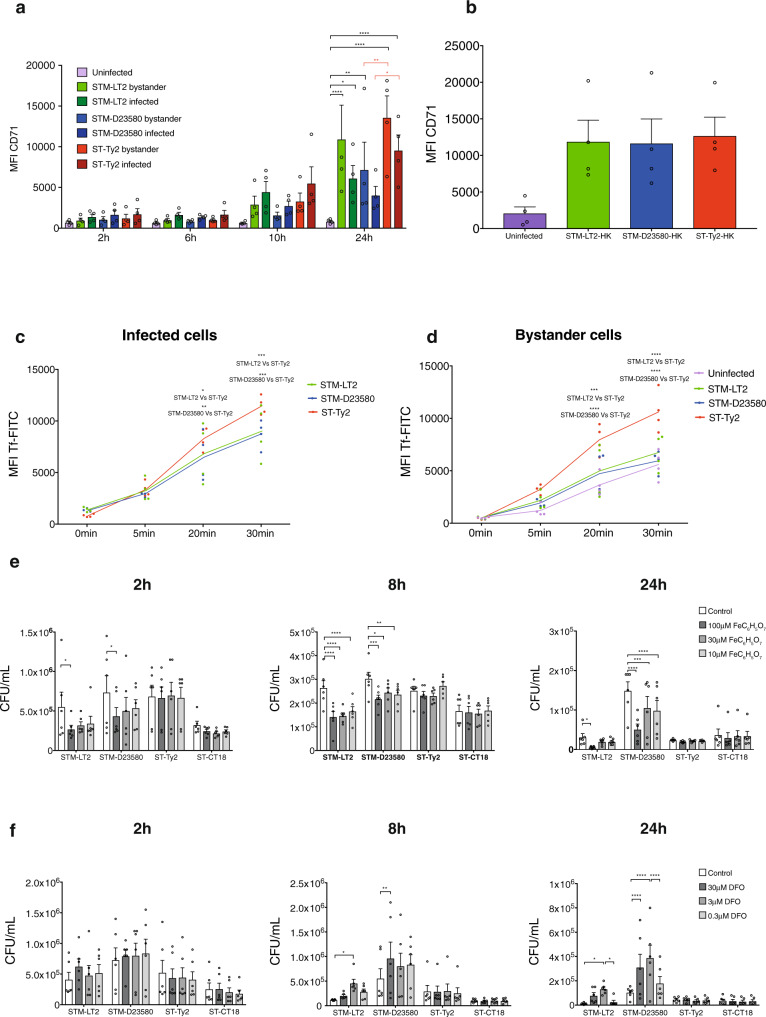
MoDCs remodel iron homoeostasis in response to *S*. Typhi*.* **a** Surface expression of TFRC measured by flow cytometry over 24 h in MoDCs infected with STM-LT2, STM-D23580 or ST-Ty2 or left uninfected. Mean ± SEM from four independent experiments are shown. Two-way ANOVA, *P* value <0.05 (*), <0.01 (**), <0.001 (***). **b** Surface expression of TFRC measured by flow cytometry on MoDCs stimulated with heat-killed (HK) bacteria for 24 h. Mean ± SEM from four independent experiments are shown. One-way ANOVA test. Flow cytometry analysis of the transferrin– FITC internalisation assay in infected (**c**) or bystander (**d**) MoDCs. Mean ± SEM from four independent experiments are shown. Two-way ANOVA, *P* value <0.05 (*), <0.01 (**), <0.001 (***). MoDCs pre-treated with FeC_6_H_5_O_7_ (**e**) or the iron chelator Deferoxamine (DFO) (**f**) were infected with STM-LT2, STM-D23580, ST-Ty2 or ST-CT18. Bacterial CFU was determined at 2 h, 8 h and 24 h p.i. White bars indicate untreated MoDCs used as controls. Mean ± SEM from six independent experiments are shown. Two-way ANOVA, *P* value <0.05 (*), <0.01 (**), <0.001 (***).

Transferrin receptor (*TFRC*) mRNA in MoDCs was upregulated by all the strains relative to uninfected cells, but its induction was significantly higher in cells infected with ST-Ty2 and STM-D23580 at 6 h p.i. We explored this observation further by examining TFRC protein surface expression at different time points over the course of infection. While all three strains induced surface TFRC expression this was significantly greater in MoDCs infected with ST-Ty2 as compared to STM-D23580 infected cells (Fig. 4a). Surprisingly by 24 h p.i. no differences in *TFRC* mRNA levels were observed among the cells infected with any of the three strains (Supplementary Fig. 6a). We believe this implies a decoupling of TFRC expression from *TFRC* mRNA transcription, specifically in MoDCs infected with STM-D23580 where high mRNA does not reflect high TFRC protein (Fig. 4a).

Interestingly, no differences were observed in the surface expression of TFRC when MoDCs were stimulated with heat-killed bacteria, indicating that live *STM-D23580* mediate this process (Fig. 4b). Notably, TFRC can be downregulated by the STM-D23580 upregulated ubiquitin ligase MARCH1^35^.

TFRC-mediated endocytosis of transferrin iron is the main mechanism of mammalian iron uptake. Consistent with their elevated surface expression of TFRC, both ST-Ty2-infected and ST-Ty2 bystander cells internalised higher levels of fluorescently labelled transferrin when compared with MoDCs challenged with either STM-D23580 or STM-LT2 (Fig. 4c, d). This data suggests that infection with *S*. Typhi drives a higher rate of iron uptake in MoDCs, whilst iron uptake is relatively suppressed in STM-D23580 infected cells.

Given the induction of iron uptake by infection, we hypothesised that iron might play a role in the antibacterial response of MoDCs to different *Salmonella* serovars. Treatment of MoDCs with iron citrate (FeC_6_H_5_O_7_), prior to and during infection, induced cellular iron loading, as indicated by reduced TFRC expression (Supplementary Fig. 6b). Furthermore, iron loading reduced recovery of viable *S*. Typhimurium from MoDCs, but unexpectedly, modifying MoDC iron status had no effect on the intracellular growth of *S*. Typhi (Fig. 4e). We then investigated the effect of iron depletion, by treating MoDCs with the extracellular iron chelator deferoxamine (DFO) for 24 h prior to and during infection. Such treatment rendered the cells iron deficient, as confirmed by increased TFRC surface expression (Supplementary Fig. 6b). Iron deficiency increased the recovery of viable *S*. Typhimurium from MoDCs, consistent with our hypothesis that DC iron uptake facilitates host control of *S*. Typhimurium infection (Fig. 4f). Notably, iron loading and deprivation had no effect on MoDC viability (Supplementary Fig. 7a, b).

### Transcriptional rewiring of *S*. Typhi in response to intracellular ROI and RNI

Inside the *Salmonella* containing vacuole (SCV) *Salmonella* is exposed to several stressors, including increased levels of ROI and RNI. The ability of myeloid cells to increase ROI and RNI production is an important defence mechanism by which intracellular *Salmonella* infection is controlled^36^, however how dual-RNA seq suggested there may be strain-specific differences in host oxidative stress responses (Fig. 3b and Supplementary Data 3 and 5). Iron plays an important role in oxidative and nitrosative stress, as an essential co-factor for the enzymes NADPH oxidase and NOS2, which produce ROI and RNI, respectively^37,38^. Free iron can also catalyse ROI production via the Fenton reaction^39^. We hypothesised that the increased ability of MoDCs pre-treated with iron to kill NTS may be due to increased production of ROI or RNI.

**Fig. 5:**
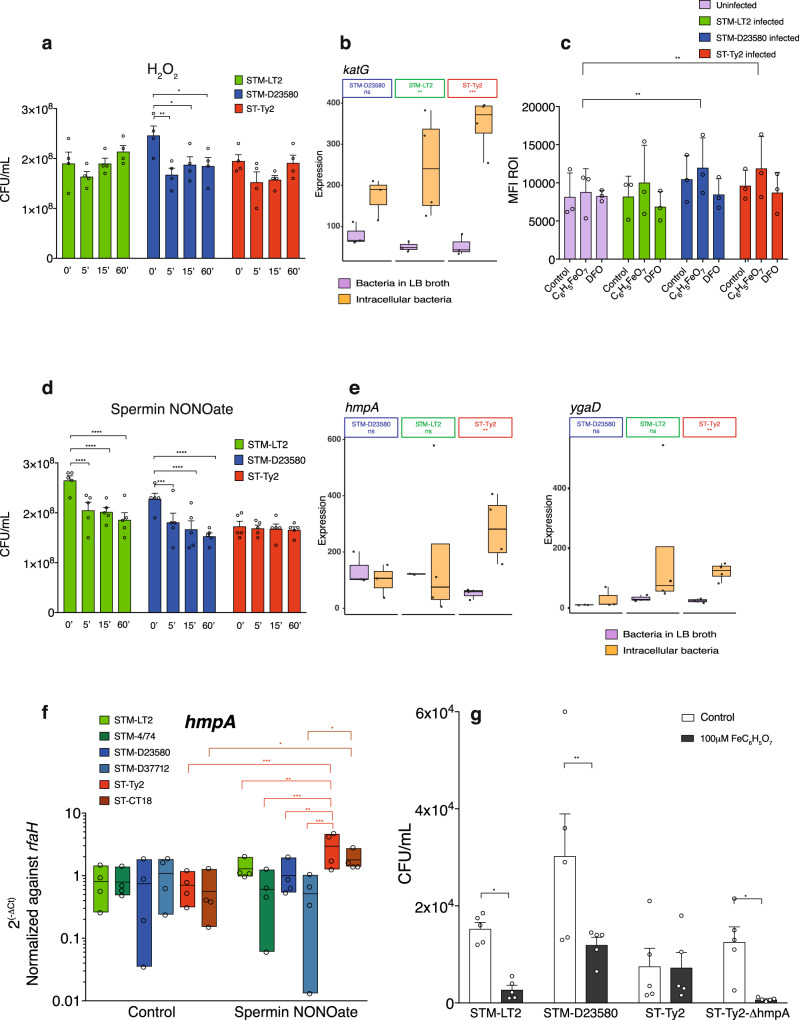
Stress-resistant mechanisms employed by *S*. Typhi during infection of MoDCs. **a** *Salmonella* surviving after challenge with 0.1 mM hydrogen peroxide (H_2_O_2_) for 5, 15 or 60 min. The mean ± SEM from four independent experiments is shown. Two-way ANOVA test, *P* value <0.05 (*), <0.01 (**). **b** Box plot displaying the normalised gene expression level of *katG* (*P* value <0.01) in intracellular bacteria or liquid culture controls. **c** Flow cytometry analysis of ROI production in MoDCs infected pre-treated with FeC_6_H_5_O_7_ or the iron chelator DFO and infected for 6 h with *Salmonella* strains. Mean ± SEM from three independent experiments are shown. Two-way ANOVA, *P* value <0.01 (**). **d** *Salmonella* surviving after challenge with 0.5 mM Spermin NONOate for 5, 15 or 60 min. The mean ± SEM from five independent experiments is shown. Two-way ANOVA test, *P* value <0.001 (***), <0.0001 (****). **e** Box plots displaying the normalised gene expression level of *hmpA* and *ygaD* (*P* value <0.01) in intracellular bacteria or liquid culture controls. **f** Box plots showing gene expression of *hmpA* detected by qPCR in multiple *Salmonella* strains after challenging with 0.5 mM Spermin NONOate for 5 min. Mean ± SEM from four independent experiments are shown. Two-way ANOVA test, *P* value <0.05 (*), <0.01 (**), <0.0001 (****). **g** MoDCs pre-treated with FeC_6_H_5_O_7_ were infected with STM-LT2, STM-D23580, ST-Ty2 or ST-Ty2 ΔhmpA. Bacterial CFU were determined at 24 h p.i. White bars indicate untreated MoDCs used as controls. Mean ± SEM from five independent experiments are shown. Two-way ANOVA, *P* value <0.05 (*), <0.01 (**).

To first test the strain-specific resistance of *Salmonella* to ROI we investigated the survival of the three bacterial serovars exposed to H_2_O_2_ in vitro. While both STM-LT2 and ST-Ty2 were able to survive and recover to stress induced by H_2_O_2_, STM-D23580 confirmed its susceptibility to ROI^40^ (Fig. 5a). Interestingly, our Dual RNA-seq data demonstrated that, unlike the H_2_O_2_-resistant STM-LT2 and *S*. Typhi, the H_2_O_2_-sensitive STM-D23580 failed to increase expression of the H_2_O_2_ detoxifying gene *katG* upon intracellular growth (Fig. 5b). Pre-treatment of MoDCs with iron citrate increased ROI production by cells infected with the invasive strains (Fig. 5c), therefore we hypothesise that iron citrate facilitates control of STM-D23580 in part through ROI production, whereas *S*. Typhi is resistant to iron facilitated ROIs production. These data also suggest iron may not facilitate control of intracellular STM-LT2 via ROI.

We then examined bacterial resistance to nitrosative stress by treating bacteria with the NO generator Spermine NONOate in vitro. Compared to ST-Ty2, the two Typhimurium strains showed a significant reduction in CFU when exposed to nitric oxide (NO) (Fig. 5d), supporting the hypothesis that *S*. Typhi may have a better capacity to resist RNI. Leveraging our dual RNA-seq dataset we found *S*. Typhi displayed increased expression of *ygaD*, involved in nitric oxide signalling^41^ and the flavohemoglobin *hmpA*, which contributes to NO-detoxifying^42,43^ (Fig. 5e and Supplementary Data 2). HmpA is expressed by the bacteria within the intracellular environment of professional phagocytes^44^ and participates in the inducible anti-nitrosative response of *Salmonella* by denitrosylating NO to NO_3_^−^ (see ref. ^42^). We measured *hmpA* expression in multiple *Salmonella* strains following exposure to Spermine NONOate. A significantly higher expression of *hmpA* was detected in *S*. Typhi strains as compared with *S*. Typhimurium stains and unstimulated bacteria (Fig. 5f).

We speculated that higher expression of *hmpA* in intracellular *S*. Typhi might contribute to its resistance to an iron-dependent antibacterial mechanism. To test our hypothesis, we constructed a *S*. Typhi mutant strain deficient to express the *hmpA* gene. The mutant strain was strongly affected by exposure to Spermin NONOate but not to H_2_O_2_ (Supplementary Fig. 8a, b), confirming the specific role of HmpA in the bacterial NO detoxing pathway. We then infected MoDC pre-treated with 100 μM of iron citrate with the bacteria strains and observed a drastic reduction of ST-ΔhmpA colonies at 24 h p.i., supporting the hypothesis that this gene may play a role in the Typhoidal resistance mechanisms against iron-induced nitrosative stress (Fig. 5g).

### Regulation of the *Salmonella* iron acquisition system upon infection of MoDCs

Our results support a role for iron in host cell antimicrobial activities, however iron is also an essential micronutrient for bacteria. For this reason, we searched our Dual-RNA-seq data to evaluate how the different *Salmonella* strains remodelled their iron homoeostasis during infection.

The intracellular bacterial transcriptome reflects the microenvironment encountered by the bacteria in the host cell vacuole^45^. We observed that all *Salmonella* strains switched on (i) the expression of metal-uptake systems, including the *sitA* and *sitB* genes, responsible for manganese and iron transport^46^, and (ii) the genes responsible for the transport of magnesium (*mgtCB*), iron mobilisation (*bfd*), and biogenesis of iron–sulfur cluster containing proteins (*sufAB*) This pattern may reflect the relatively low levels of magnesium, manganese and iron within the SCV. Particularly, STM-D23580 displayed an increased expression of iron acquisition systems such as *feoAB*, required for the acquisition of Fe^2+^ in anaerobic condition^47^, and the *cirA/iroN* siderophore import system (Supplementary Fig. 9a and Supplementary Data 2). Gene set enrichment analysis confirmed the increased expression of siderophore dependent iron acquisition system in the invasive STM-D23580 (Supplementary Fig. 10 and Supplementary Data 7).

Interestingly, intracellular *S*. Typhi did not show particularly high expression of genes involved in iron acquisition, consistent perhaps with the high level of iron uptake in ST-Ty2-infected MoDCs. However, intracellular *S*. Typhi did show particular induction of the non-ribosomal protein synthetase *entF*, and the siderophore synthesis gene *entC*^48^ (Supplementary Fig. 9a and Supplementary Data 2). Since EntF has been shown to confer resistance to H_2_O_2_ in *Salmonella*^49^, we hypothesised that *S*. Typhi siderophore production maybe be linked to its resistance to oxidative and nitrosative stresses.

To test our hypothesis, we measured siderophore production in supernatants from bacterial cultures by implementing the chrome azurol sulphonate (CAS) assay^50^. As expected^51^, growth in iron-depleted minimal medium (MM) enhanced siderophore activity relative to rich LB (Supplementary Fig. 9b, c), however exposure to 100 μM of H_2_O_2_ or 250 μM of Spermin NONOate suppressed *S*. Typhimurium siderophore production, whilst *S*. Typhi maintained siderophore production unperturbed by ROI or RNI exposure (Supplementary Fig. 9b, c). We hypothesise that *S*. Typhi’s ability to sustain siderophore production might constitute another defence mechanism against ROI and RNI stresses experienced in vivo.

### Strain specificity to iron-dependent killing is recapitulated in human gut tissue

**Fig. 6:**
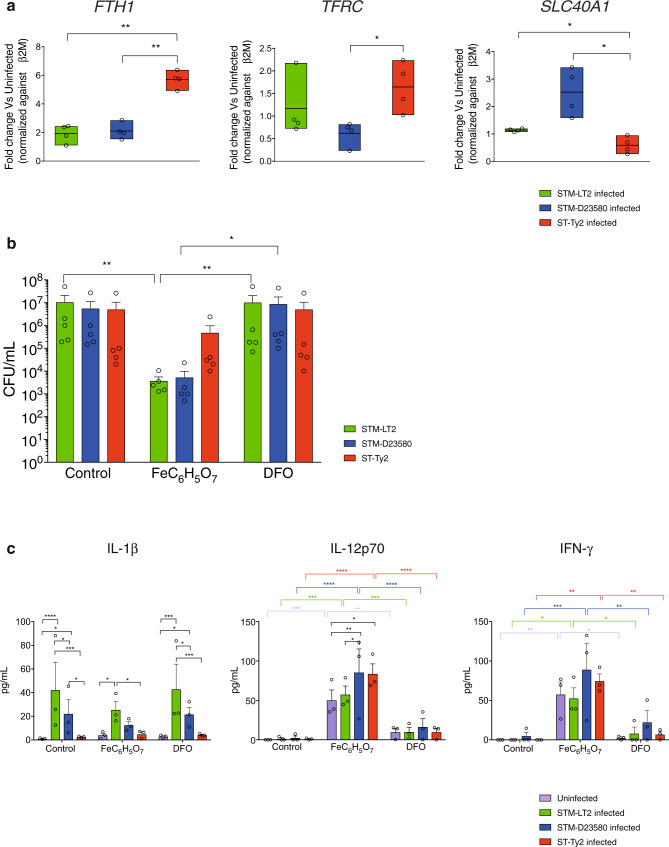
Ex vivo validation. **a** Gene expression of *FTH1, TFRC* and *SLC40A1* measured by qPCR at 4 h p.i. in Terminal Ileo biopsies infected with *Salmonella* strains. Mean ± SEM from four independent experiments are shown. Two-way ANOVA test, *P* value <0.05 (*), <0.01 (**). **b** Terminal Ileo biopsies pre-treated with FeC_6_H_5_O_7_ or the iron chelator Deferoxamine (DFO) were infected with STM-LT2, STM-D23580 or ST-Ty2. Bacterial CFU were determined at 4 h p.i. Mean ± SEM from five independent experiments are shown. Two-way ANOVA, *P* value <0.05 (*), <0.01 (**). **c** Terminal Ileo biopsies pre-treated with FeC_6_H_5_O_7_ or the iron chelator Deferoxamine (DFO) were infected with STM-LT2, STM-D23580 or ST-Ty2 and IL-1β, IL-12p70 and IFN-γ were quantified by ELISA at 4 h p.i. The mean ± SEM from three independent experiments is shown. Two-way ANOVA test, *P* value <0.05 (*), <0.01 (**), <0.001(***), <0.0001(****).

*Salmonella* colonise and invade the distal ileum of humans^52^. We asked whether the results observed in isolated MoDCs could be replicated in the cells that the bacteria encounter in the lamina propria during infection^53^. To this purpose, terminal ileum biopsies were infected with the three *Salmonella* strains or left uninfected. *S*. Typhi-infected ileal cells expressed significantly higher levels of *TFRC*, *FTH1* and lower level of iron exporter ferroportin (*SLC40A1*) (Fig. 6a) consistent with the enhanced iron uptake and storage phenotype observed in *S*. Typhi-infected MoDCs.

To test our hypothesis that iron pre-loading may improve cell-intrinsic immunity and cellular bactericidal mechanisms, human terminal ileum biopsies were treated with iron citrate prior to and during infection with *Salmonella* strains. As observed for MoDCs, pre-treatment with iron citrate reduced recovery of viable *S*. Typhimurium, but not *S*. Typhi (Fig. 6b).

Infection of the ileal biopsies provides a unique chance to explore how iron availability might modulate the multi-cellular immune response to *Salmonella* infection. We measured the production of IL-1β, IL-12p70 and IFN-γ at 2.5 h p.i. following iron citrate or DFO pre-treatment. Interestingly, we observed that iron loading induced a significantly higher production of both IL-12p70 and IFN-γ in each condition, suggesting that iron might facilitate activation of myeloid cells and lymphocytes^54^. In addition, the supernatants of cells infected with invasive *Salmonella* strains contained significantly higher levels of IL-12p70 as compared to uninfected or STM-LT2-infected cells. (Fig. 6c). The production of IL-1β was significantly higher in cells infected with the Typhimurium strains as compared to Typhi, confirming that Typhi suppresses some aspects of the inflammatory response in the intestine^55,56^. Iron treatment did not alter IL-1β production, arguing against non-specific immune activation by iron (Fig. 6c).

Altogether, our findings suggest that infection of MoDCs recapitulate the predominant steps of *Salmonella* infection in immune cells in the human terminal ileum.

## Discussion

**Fig. 7:**
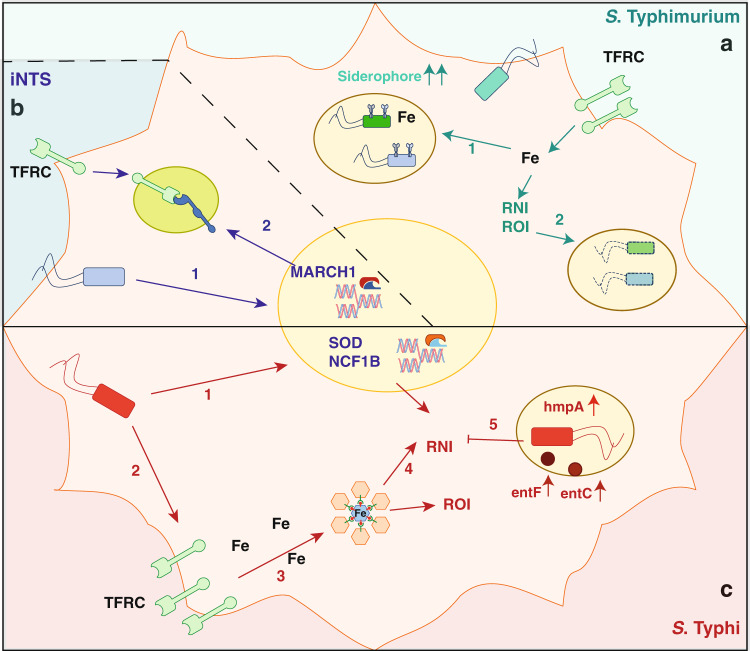
Strategies adopted by *Salmonella* to prevent iron-driven bactericidal mechanisms. **a** *S*. Typhimurium overexpress different iron acquisition systems to compete for the intracellular iron (1). Excess of iron can be used by the host to generate antimicrobial mechanisms which improve bacterial clearance (2). **b** Non-typhoidal *Salmonella* limit intracellular iron loading by reducing surface expression of TFRC most likely through the action of MARCH1 (1 and 2). **c** Infection with *S*. Typhi results in increased iron loading and activation of oxidative and nitrosative stress mechanisms (1–4). However, the higher production of detoxifying enzymes, such as HmpA (5), protect the bacteria by the toxic effect of RNI. Enterobactin (entF and entC) are responsible of iron acquisition during stress (5).

We employed a dual RNA-seq approach to achieve unique insights into the paired transcriptional responses of host and pathogen during infection of human DCs with different invasive *Salmonella* pathovariants. Overall, we observed that different *Salmonella* strains adopt different strategies to prevent the host iron-driven antimicrobial defence. *S*. Typhimurium overexpress different iron acquisition systems to compete for the intracellular iron that might otherwise be used to generate stress response resulting in bacterial clearance (Fig. 7a). Invasive NTS attempt to reduce intracellular iron loading by limiting surface expression of TFRC most likely through the action of MARCH1, requiring them to increase their iron acquisition even further (Fig. 7b). In contrast, *S*. Typhi permits MoDCs iron uptake to continue unperturbed as it appears to be resistant to iron-dependent bacterial defences, perhaps in part due to its enhanced induction of RNI defence genes, including *hmpA*, upon infection (Fig. 7c). In parallel, we provide evidence that invasive non-typhoidal *Salmonella* employs several distinct mechanisms targeting more classic aspects of immunity to impair DC function.

Intracellular pathogens like *Salmonella* compete with the host to obtain essential nutrients, such as iron. Iron starvation or ‘nutritional immunity’ has been proposed to be an important defence mechanism against intracellular bacteria in macrophages^34,57,58^; however little is known about the role iron plays during infection of other myeloid cells. Iron is central to cellular biochemistry^59^ and therefore an interesting trade-off exists between depriving the pathogen of iron and the requirement of iron for host cell immunity. The outcome of this trade-off is likely to depend on the type of host cell and species/strain of pathogen.

We observed that upon *S*. Typhi infection, MoDCs remodel iron trafficking, strongly increasing iron uptake (*TFRC*, *STEAP3*), storage (*FTH)*, and iron delivery to the mitochondria, both for heme and Fe–S cluster synthesis (*SLC25A37* (mitoferrin)). The specific *S*. Typhi effectors promoting this MoDC iron phenotype are unknown, but the increased expression of *STAT6* and *IL4R* we observe in ST-Ty2-infected cells may contribute, as STAT6 signalling and type 2 cytokines are known to promote TFRC-mediated iron uptake and intracellular storage by ferritin in macrophages^60^. The particular ability of *S*. Typhi to promote a type II immune phenotype in dendritic cells and control iron metabolism deserves further investigation.

The lowest TFRC protein expression and TF uptake was reliably observed in STM-D23580-infected cells, suggesting that STM-D23580 may actively suppress of host cell iron uptake. This may be a virulence mechanism to avoid iron-mediated host defence mechanisms as supplementation with iron citrate, an iron source that can be taken up independently from the transferrin receptor^61^, improved MoDC control of *S*. Typhimurium infection, whereas iron deprivation further enhanced intracellular *S*. Typhimurium growth.

However iron is an essential micronutrient for *Salmonella* growth and like all successfully adapted pathogens, *Salmonella* has developed sophisticated mechanisms to compete with host iron-scavenger proteins for iron acquisition, transport and storage^58^. In particular, STM-D23580 displayed an increased expression of all the main iron acquisition systems. This finding may support the hypothesis that, as a consequence of relatively lower expression of TFRC and iron acquisition by STM-D23580-infected MoDCs, which may in fact be driven by STM-D23580 itself, STM-D23580 experiences a poorer iron microenvironment in the SCV and must therefore mount a more robust iron acquisition response to support growth.

Intriguingly, as TFRC is reported to be targeted for degradation by MARCH1^62^, we speculate that the observed lower surface expression of TFRC in STM-D23580, may be due to the constitutive expression of *MARCH1* by MoDCs infected with this strain^9^. Therefore, in addition to preventing antigen presentation, elevated *MARCH1* in STM-D23580-infected cells may facilitate evasion of iron-mediated host defence. It has been reported that the *S*. Typhimurium effector SteD causes MARCH8-dependent ubiquitination and depletion of surface MHC-II in murine dendritic cells and cell line and thus impairs Antigen presentation and T-cell activation^63^. Uncovering the STM-D23580-specific effectors which maintain heightened MARCH1 activity in infected human immune cells will be an important future line of enquiry.

In contrast, we propose *S*. Typhi permits MoDC iron uptake to continue unperturbed, as it is resistant to DC iron-dependent bacterial control, perhaps in part due to the enhanced induction of genes which detoxify ROI and RNI^64^. Catalases detoxify H_2_O_2_ by catalysing its decomposition to O_2_ and H_2_O, while the flavohemoprotein HmpA contributes to NO detoxification catalysing the conversion of NO to relatively inert nitrate (NO^3−^)^42^. We observed induction of *katG* and *hmpA* genes in intracellular *Salmonella* and after in vitro challenge with NO consistent with published literature^44^. However, this transcriptional response was particularly profound for *S*. Typhi and we therefore suggest that heightened expression of *katG* and *hmpA* and better tolerance of H_2_O_2_ and NO may support the invasiveness of *S*. Typhi. In contrast our data suggest STM-D23580 is particularly sensitive to ROI and RNI which may underscore its drive to suppress MoDC iron uptake.

Furthermore, when grown in conditions mimicking the ROI or RNI shock, we find that *S*. Typhi successfully sustained siderophore production, compared with the two Typhimurium strains. Catecholate siderophores have been reported to have an antioxidant role in addition to their well-known role in iron chelation^65^. Therefore, we propose the significantly higher expression of *entF* in *S*. Typhi may hint to altered regulation of siderophore production in the Typhoidal strain, which may facilitate resistance to oxidative stress and its invasive phenotype.

Mouse models are an indispensable tool to study the pathology of human disease and allow the identification of several factors that can be targeted for treatment or vaccines in humans. However, not all human infections are replicable in animals. Although the infection with *S*. Typhimurium in mice has been utilised as a model to study typhoid-like disease, due to the characteristics and genetic divergence between both *S*. Typhimurium and *S*. Typhi^66^ it is not an optimal model to analyse the nuances of host–pathogen interactions. To understand how broadly applicable our in vitro results in MoDCs are, we infected human terminal ileum biopsies with *Salmonella* species. The small intestine is the natural site of infection and, in this model, bacteria infect tissue myeloid cells, which are known to have unique phenotypes compared to macrophages or DCs derived in vitro from circulating monocytes^67,68^.

Our data suggested that, similar to MoDCs, ileum cells infected with *S*. Typhi expressed higher levels of iron uptake and storage machinery compared to *S*. Typhimurium species, while limiting iron export. Consistent with a model where non-typhoidal *Salmonella* species are particularly sensitive to iron-mediated defence mechanisms and so limit iron uptake by DCs, iron supplementation greatly reduced the CFU of *S*. Typhimurium but not *S*. Typhi. Iron supplementation also increased the concentration of IL-12p70 and IFN-γ by infected biopsies suggesting that cellular iron levels may regulate cytokine production and protective type I immune responses in the gut. How iron availability modulates immune cell–cell interactions in the human gut and what this means for infections is beyond the scope of the current study but will be an interesting future research direction.

One important unaddressed question in *Salmonella* biology is why co-infection with malaria predisposes specifically to disseminated infection with NTS such as STM-D23580, but not *S*. Typhi infection^65,69^. Malaria is well established to perturb host systemic iron metabolism^70^ and can drive functional systemic iron deficiency through raised hepcidin^71^. Our results suggest that the further undermining of iron-dependent host immune defences may be one mechanism by which malaria infection increases susceptibility to iNTS and warrants further investigation.

Invasive strains of non-typhoidal *Salmonellae* have emerged as a major cause of bloodstream infection showing resistance to multiple antibiotics^3^. We have previously demonstrated that the invasive non-typhoidal *Salmonella* D23580 is able to impair human DC antigen presentation functions through the modulation of MARCH1/IL10 axis, as well as evade MAIT cell activation^9,72^. Here, we suggest that STM-D23580 may modulate the IL-21 and TLR8-dependent RNA sensing and IFN signalling as strategy to impair the host response. IL-21 inhibits DC functions and induces apoptosis of conventional DCs^73,74^. A recent study revealed an interplay between IL-21 and type I IFN in the innate immune response to Methicillin-resistant *Staphylococcus aureus* (MRSA). Basal MRSA clearance was enhanced when IL-21 signalling was blocked, due to increased type I interferon production. Overall, these observations suggest that the D23580 pathovar might employ several strategies to impair MoDC functions.

Our dual RNA-seq data highlighted a dynamic adaptation of iron metabolism in both host and pathogen during infection. In contrast to the paradigm of nutritional immunity in macrophages, we find that MoDCs actively increase iron uptake during infection and employ iron-dependent mechanism of host defence, delving into these mechanisms in greater detail will be an exciting future research direction. Despite the observation that invasive STM-D23580 demonstrates genome degradation resembling that of the human-restricted pathogen *S*. Typhi^3^, we find that the two invasive strains take unique approaches to evade host immunity and in particular host MoDC iron-dependent defences. Given the involvement of iron in the control of microbial pathogenicity, it appears reasonable to propose that targeting iron may offer therapeutic value; however, our work highlights how different approaches may need to be taken for different serovars.

## Methods

### Bacterial strains and labelling

*Salmonella enterica* serovar Typhimurium (STM) strain LT2 (ATCC 700220) and the clinical isolate STM-D23580, were used as representative non-typhoidal *Salmonella* sequence type 19 (ST19) and type 313 (ST313), respectively. Strain LT2 is one of the principal *Salmonella* laboratory strains used in cellular and molecular biology^75^. Strain D23580 was isolated from the blood of an HIV-negative Malawian child with malaria and anaemia. *Salmonella enterica* serovar Typhi (ST) strain Ty2 (ATCC 700931) was used as representative typhoidal serovar. *Salmonella enterica* serovar Typhi strain CT18 is an emerging multidrug-resistance serovar isolated from a Vietnamese child who was suffering from typhoid fewer^76^. *Salmonella enterica* serovar Typhimurium strain 4/74 and D37712 were kindly provided by Prof. Jay Hinton. All bacteria were grown to logarithmic growth phase in LB Lennox broth (Sigma) supplemented with sucrose (Sigma) at a final concentration of 10%. Aliquots were kept frozen at –80 °C until use, while bacterial viability was monitored periodically. For each experiment, an aliquot of bacteria was thawed, diluted in RPMI 1640 (Sigma) and incubated in the presence of 5 uM of CellTrace^TM^ Far Red Cell Proliferation kit (ThermoFisher) at 37 °C for 20 min while shaking (200 rpm). The CellTrace^TM^ Far Red Cell Proliferation kit had no effects on bacterial viability and invasion (Supplementary Fig. 11a, b). Bacteria were then washed and resuspended in RPMI to obtain a multiplicity of infection (MOI) of 10:1. The number of microorganisms was assessed at each time point post infection by plating tenfold dilutions of the bacterial suspension, in quadruplicate, on LB Lennox agar (Sigma). The number of bacteria was determined as colony-forming units (CFU).

### Generation of the *S*. Typhi ΔhmpA strain

Bacteria deficient for *hmpA* expression were generated using λ-red recombineering. A triple STOP codon (TAGaTGAaTAA) was inserted into the *hmpA* coding sequence 51 nucleotides after the transcription start site, and selection of successfully integrated colonies was facilitated by a co-inserted em7-Kanamycin resistance cassette. The cassette was PCR-amplified and integration was achieved using 50 nt micro-homology arms as previously described^77^. After the recombineering reaction, successfully recombined bacteria were selected for on Kanamycin containing LB plates. Individual clones were screened for the presence of the STOP cassette using PCR primers outside and inside the cassette for both arms. All clones were verified to contain the functional STOP by Sanger sequencing.

### Preparation of heat-killed bacteria

*Salmonella* strains were grown in LB broth supplemented with 10% sucrose. Bacteria were harvested during the logarithmic growth phase, diluted to an OD 0.5, and killed by heating at 100 °C for 15 min. The suspension of heat-killed (HK) bacteria was allowed to cool at room temperature (RT) for 15 min and, subsequently, plated on LB agar to confirm bacteria were no longer viable.

### Generation and infection of MoDCs

Leukocyte Reduction System cones were obtained from the UK National Blood Centre with informed consent following local ethical guidelines. Blood was diluted in phosphate-buffered saline (PBS) and layered on a standard density gradient (Lymphoprep^TM^). Peripheral blood mononuclear cells (PBMC) were collected from the interface and washed in PBS at 4 °C. Monocytes were obtained by the magnetic-positive selection, using the human anti-CD14^+^ MicroBeads (Miltenyi Biotec, Germany) according to the manufacturer’s protocol.

Freshly isolated monocytes were plated in tissue culture-treated dishes (Falcon) at a density of 1E+06/mL. Differentiation into monocyte-derived dendritic cells (MoDCs) was induced in the presence of 40 ng/mL of recombinant human (rh) granulocyte macrophage-colony-stimulating factor (GM-CSF; PeproTech) and 40 ng/mL rh Interleukin-4 (IL-4; PeproTech).

After 5 days of culture, MoDCs were harvested, resuspended in RPMI supplemented with 10% foetal bovine serum (FBS, Sigma) and 2 mM l-glutamine (Sigma) at a density of 1E+06/mL, and seeded 1 mL/tube in polypropylene tubes (Falcon). MoDCs were infected with STM-LT2, STM-D23580 or ST-Ty2 at a MOI of 10 and immediately spun down for 5 min to maximise bacteria–cell contact. This MOI was used, so that the frequency of infection was high enough to enable observation of infection events while minimising *Salmonella*-induced MoDCs death.

After incubation for 45 min at 37 °C, extracellular bacteria were washed away with PBS. MoDCs were then incubated for 30 min in RPMI supplemented with 10% FBS, 2 mM l-glutamine and 100 µg/mL of gentamycin (MP Biomedicals) to kill extracellular bacteria. Gentamycin concentration was subsequently reduced to 30 µg/mL for the remainder of the experiment.

### Collection of tissue biopsies and infection

All protocols for recruitment of human subjects and use of human terminal ileum biopsies were approved by NHS National Research Ethics Service (NRES) research ethics committee (REC) references for the study include 18/WM/0237. Protocol number: 13463. IRAS project ID: 243653. Following informed consent, biopsies were collected from volunteers without chronic medical conditions who were scheduled to undergo a routine colonoscopy and were screened for good general health. Biopsies were transported to the laboratory and stored by freezing in 1 ml of Cryostor DS10 (Sigma Aldrich) to be processed simultaneously. Viability was similar to those of freshly isolated samples.

On the day of the infection, biopsies were defrosted and incubated in an EDTA-enriched dissociation medium (HBSS (ThermoFisher), 1% penicillin–streptomycin (Life Technologies), 1 M HEPES (Life Technologies), 5 mM EDTA (Invitrogen), 2 mM dithiothreitol (Invitrogen), 1% FBS) at 37 °C for 80 min with agitation to remove the epithelial layer from the underlying lamina propria. The remaining bites of biopsy were subject to enzymatic dissociation with the Lamina Propria dissociation kit (Miltenyi). Cells were resuspended at a density of 1E+06/mL in RPMI supplemented with 10% FBS, 2 mM l-glutamine and 0.1 mM non-essential amino acid (Sigma) and seeded 250 µL/well in a 96-well plate. Cells were then infected with STM-LT2, STM-D23580 or ST-Ty2 at a MOI of 5 or left uninfected and immediately spun down for 5 min to maximise bacteria–cell contact. At 2 h p.i., 100 μg/mL gentamicin was added for 30 min. Cells were then washed and resuspended in a fresh medium containing 30 μg/mL gentamicin for the remaining of the experiments.

### Flow cytometry sorting

At 6 h p.i., cells were harvested, washed, stained with Propidium Iodide (Biolegend) and resuspended in FACS buffer containing PBS, 0.3% (v/v) bovine serum albumin (BSA, Sigma) and 2 mM EDTA (Invitrogen). Samples were immediately acquired on a MoFlo sorter (Beckman Coulter) applying fluorescence minus one control to adjust compensation and sorting gates. The accuracy of a high purity single-cell sorting was confirmed using beads and cells. In total, 20,000 live single cells were sorted into Eppendorfs containing 10 µL of PBS supplemented with 40 U/µL of RiboLock RNase inhibitor (ThermoFisher), spun down, and immediately processed for RNA extraction.

### Quantification of intracellular bacteria

To quantify the replication of intracellular bacteria, infected host cells were washed with PBS, resuspended in FACS buffer, stained and FACS sorted as described before. For each experiment, ten single cells per experimental condition were sorted in eight-well strips containing 1% saponin (Sigma) in PBS and plated onto agar plates to determine the number of intracellular CFU.

### RNA extraction and library preparation

Sorted cells were spun down and the pellet resuspended in Monarch RNA lysis buffer (New England BioLabs) supplemented with 2 mg/mL of Lysozyme (ThermoFischer). Bacteria grown in LB broth and harvested at the mid-logarithmic phase were used as control. Total RNA extraction was performed with the Monarch^®^ Total RNA Miniprep Kit (New England BioLabs) according to the manufacturers. RNA quality and quantity were assessed using a high-sensitivity RNA ScreenTape assay in a 4200 TapeStation (Agilent Technologies).

Libraries were prepared by using the SMARTer^®^ stranded total RNA-seq v2 pico input Mammalian (Takara) with minor technical adaptations. PCR1 was carried on for 5 cycles and PCR2 for 11 cycles (for the bacterial libraries) or 12 cycles (for the host–pathogen libraries). After cDNA synthesis, the human ribosomal cDNA was removed by using probes specific to mammalian rRNA.

Quality was assessed with a high-sensitivity DNA chip in a 4200 TapeStation. Finally, 750 ng of the resulting libraries were used for the enrichment protocol. Libraries were pooled in equimolar amounts. Sequencing was performed in the paired-end mode for 2 × 150 bp cycles using the Hiseq4000 and the NovaSeq6000 or the MiSeq sequencers (Oxford Wellcome Centre for Human Genetics) for dual RNA-seq or bacteria-only samples, respectively.

### SureSelect custom target enrichment library preparation

The SureSelect custom capture library probes were designed for the capture of cDNA sequences from *Salmonella* as follows. First, multiple sequence alignment of the genomes of all three *Salmonella* strains was carried out and visualised using mauve software (version 2.4.0)^78^ and all orthologs were mapped between the three strains. Where an ortholog was found between two or more strains, in each case sequences were re-aligned using R package 'msa'^79^ and consensus matrix was checked for single-nucleotide substitutions and small indels. Briefly, using a sliding window (20 nt width) approach, we computed sequence divergence for each window and the window was considered as conserved if it differed in fewer than 20% of all positions. Contiguous bins passing these criteria were merged and if they were at least 120 nt in length, and had the overall divergence as <10%, they were put forward for targeting. In cases where <90% of all bases in the shortest ortholog could be thus assigned, sequences of all orthologs were targeted individually. Similarly, strain-specific loci were put forward for targeting individually. Furthermore, we filtered out ribosomal and transfer RNA genes from our panel in order to deplete these high-abundance RNAs by negative selection. The selected *Salmonella* probe-target sequences were then uploaded to SureDesign and probes were synthesised by Agilent Technologies.

The adapter-attached pooled DNA library was hybridised to the *Salmonella* capture library designed for this study. Agilent’s SureSelect^XT^ Target Enrichment protocol version C3 was followed for hybridisation and capture. Specifically, 750 ng of pooled libraries was mixed with the SureSelect ^XT^ Blocker Mix and was incubated at 95 °C for 5 min and 65 °C for 5 min. The SureSelect^XT^ Hybridisation Buffer was then added to the sample and hybridizaton was performed at 65 °C for 24 h. Immediately after the reaction the captured DNA was purified by using Dynabeads MyOne Streptavidin T1 beads (ThermoFisher Scientific). Then, the DNA library, which was attached to streptavidin beads, was amplified by PCR. The PCR cycling conditions were as follows: an initial denaturation at 98 °C for 2 min; followed by 11 cycles (for the bacterial libraries) or 16 cycles (for the host–pathogen libraries) of 98 °C for 30 s, 57 °C for 30 s, and 72 °C for 1 min; and a final extension at 72 °C for 10 min. After PCR, streptavidin beads were removed from the sample by using a magnet stand, and the PCR products, which were not associated with the beads, were further purified with Agencourt AMPure XP. The quality was assessed with a high-sensitivity DNA chip in a 4200 TapeStation.

Sequencing was performed in paired-end mode for 2 × 150 bp cycles using the NovaSeq6000 the MiSeq sequencers (Oxford Wellcome Centre for Human Genetics) for dual RNA-seq or bacteria-only samples, respectively.

### RNA-sequencing read mapping and expression quantification

Initially, sequencing quality of all libraries was assessed using fastQC software (version 0.11.9). Sequencing adapters and poor quality (<20) bases were trimmed using cutadapt software (version 1.16)^80^. *Salmonella* reference genome annotation and sequences for each strain were downloaded from NCBI. Human hg38 analysis set reference genome was downloaded from UCSC ftp site^81^ and corresponding genome annotations in GTF format were downloaded using the UCSC Table Browser tool^82^. Joint *Salmonella*-human reference genomes were indexed separately for each strain and reads were aligned against these references using STAR aligner (version 2.4.2a)^83^. Cultured bacterial libraries were also aligned against the joint human-*Salmonella* reference to avoid introducing any processing biases, although these libraries were not expected to contain any human sequences and vice versa for uninfected samples. Picard tools were used for additional QC and duplicate read marking. The Resulting BAM files were further filtered for trans-species multi-mapping reads, removing all alignments (<1%) that mapped to both human and *Salmonella* genomes. Subread featureCounts (version 1.6.2)^84^ was then used to summarise gene read counts, with the same species multimappers counted as fractions.

For comparative analyses of paired enriched-non-enriched libraries, sequencing reads from all infected samples were downsampled to 1 M and 10 M reads using SeqTK (version 1.0-r68e) and processed as described above. Libraries generated from bacteria grown in LB broth were downsampled to the lowest depth sample for each comparison. We also included bystander MoDC samples as an additional control in this analysis, where, as expected, we still recovered some bacterial sequences.

### Differential expression analysis

Counts matrices were imported into R for further processing. Library-size scaling factors were computed and data were normalised using DESeq2 R package (version 1.24)^85^. All differential expression analyses were also performed with DESeq2.

PCA analyses were carried out in R using prcomp base function, using top 1000 most variable genes as input. Data were normalised and transformed using variance-stabilising transformation prior to PCA. Some visible batch effects were observed in PCA analysis (Fig. 2b and Supplementary Fig. 2h–j) where we included samples from pilot experiment (*n* = 2 per group). These were included as a blocking variable in differential expression tests where appropriate. Gene Ontology enrichment analyses were carried out using R package clusterProfiler (version 3.12.0)^86^ using GO.db annotations for host genes. For bacterial genes, GO annotations were obtained from Uniprot.

### Fluorescence-activated cell sorting (FACS) analysis

MoDCs were stimulated with STM-LT2, STM-D23580, ST-Ty2 or left unstimulated. At specific time points post infection, MoDCs were washed and incubated for 30 min with anti-CD71 at a concentration of 1:100 (PeCy7; CY1G4, BioLegend). Zombie Aqua^TM^ fixable viability dye (BioLegend) was used for the exclusion of dead cells. After incubation, cells were washed in FACS buffer and fixed in 2% PFA. Samples were acquired on a Fortessa X20 flow cytometer (BD Biosciences) and files were analysed on Flowjo (v.10.4.1).

### Quantitative PCR

#### *SLC25A3*, *SOD2*, *IL6*, *IL10* gene expression

MoDCs were stimulated with several *Salmonella* strains or left unstimulated. At 6 h p.i. single cells were FACS sorted in lysis buffer and cDNA was prepared according to Smart-seq2 protocol as described in Picelli et al.^87^. cDNA from eight individual cells from each experimental group was pooled and qPCR for *SLC25A3, SOD2, IL6* and *IL10* was performed. qPCR reaction was carried on in 96-well plate in 20 μL final volume containing: TaqMan gene expression Master Mix (ThermoFisher), TaqMan gene expression assay (ThermoFisher, *SLC25A3*: Hs00358082_m1; *SOD2*: Hs00167309_m1; *IL6*: Hs00174131_m1; *IL10:* Hs00961622_m1) H_2_O and 10 ng of cDNA. After the initial denaturation steps at 50 °C for 2 min and 95 °C for 2 min, PCR was performed for 40 cycles (95 °C for 1 s and 60 °C for 20 s for each cycle) by using the Quanti7 Studio machine. Fold changes were determined using the 2−^ΔCt^ method. The mRNA levels were expressed in relative copy numbers normalised against the beta 2 microglobulin (*β2M*) mRNA (TaqMan gene expression assay Hs00984230_m1).

#### *TFRC*, *FTH1* and *SLC40A1* gene expression

MoDCs or Termianl Ileo biopsies were stimulated with either STM-LT2, STM-D23580 or ST-Ty2 or left unstimulated. At 24 h p.i. or 4 h p.i., cells were collected and RNA was extracted using the Monarch kit and cDNA was prepared with the High-Capacity RNA-to-cDNA kit (ThermoFisher) according to the manufactures. qPCR reaction was carried on in 96-well plate in 20 μL final volume containing: TaqMan gene expression Master Mix (ThermoFisher), TaqMan gene expression assay (ThermoFisher, *TFRC*: Hs00951083_m1; *FTH1*: Hs02596865_g1; *SLC40A1*: Hs00205888_m1) H_2_O and 10 ng of cDNA. After the initial denaturation steps at 50 °C for 2 min and 95 °C for 2 min, PCR was performed for 40 cycles (95 °C for 1 s and 60 °C for 20 s for each cycle) by using the Quanti7 Studio machine. Fold changes were determined using the 2−^ΔCt^ method. The mRNA levels were expressed in relative copy numbers normalised against the beta 2 microglobulin (*β2M*) mRNA (TaqMan gene expression assay Hs00984230_m1).

#### *hmpA* gene expression

Bacteria were grown overnight in LB broth and diluted 1:33 in PBS. After 5 min stimulation with 0.5 mM of Spermin NONOate bacteria were spun down and the pellet resuspended in Monarch RNA lysis buffer (New England BioLabs) supplemented with 2 mg/mL of lysozyme (ThermoFisher). Total RNA extraction was performed with the Monarch^®^ Total RNA Miniprep Kit (New England BioLabs) according to the manufacturers. Total cDNA was prepared with the SuperScript II (ThermoFisher) using 150 ng of random primers (ThermoFisher) according to the manufacturers.

qPCR reaction was carried on in 96-well plate in 20 μL final volume containing: Dual Lock DNA pol Master Mix (Life Technologies), forward and reverse primers (*hmpA* forward: GATACCCCCGTTTCGCTGAT, *hmpA* reverse: CGCGGTATGCTGTTCTTTCG; *rfaH* forward: AATAACGCTGGAAGGCACGA, *rfaH* reverse: CAGCGAACCGCTCTTTCCTA) at a final concentration of 1 μM each, H_2_O and 10 ng of cDNA. After the initial denaturation steps at 50 °C for 2 min and 95 °C for 2 min, PCR was performed for 40 cycles (95 °C for 3 s and 60 °C for 30 s for each cycle) by using the Quanti7 Studio machine. The specificity of primers was confirmed using Primer-BLAST (NCBI). Fold changes were determined using the 2−^ΔCt^ method. The mRNA levels were expressed in relative copy numbers normalised against the *rfaH* mRNA.

### Transferrin uptake assay

To evaluate the uptake of Transferrin following bacterial infection, cells infected with STM-LT2, STM-D23580 or ST-Ty2 or left uninfected for 24 h were rinsed and incubated at 37 °C in serum-free medium for 30 min to remove any residual transferrin.

After the cells were harvested and washed, they were incubated with 50 mg/mL 488-conjugated human transferrin (Invitrogen) in binding buffer (RPMI 1640 containing 25 mM 4-(2-hydroxyethyl)-1-piperazineethanesulfonic acid (HEPES) pH 7.4, 0.5% BSA) at 37 °C for 5 min, 20 min or 30 min. Internalisation was stopped by chilling the cells on ice for 10 min and external transferrin was removed by washing with ice-cold PBS. The fluorescence intensity of internalised transferrin was measured by flow cytometry.

### Iron depletion and iron-loading condition

MoDCs were obtained as described above. At day 4 of differentiation, cells were harvested, washed and resuspended at a density of 1E+06/mL in RPMI supplemented with 10% foetal bovine serum (FBS, Sigma), 2 mM l-glutamine (Sigma), GM-CSF, IL-4 and iron (III) citrate (Sigma) or deferoxamine mesylate salt (DFO, Sigma) at several concentrations. The following day, MoDCs were harvested, resuspended in medium supplemented with iron (III) citrate or DFO, and seeded 1E+06/mL/tube in polypropylene tubes (Falcon). MoDCs were infected with STM-LT2, STM-D23580 or ST-Ty2 as described above. At 24 h post infection cells were lysed by addition of 500 μL of saponin 1% (w/v) (Sigma) in PBS followed by 5 min incubation at 37 °C. Cell lysates were serially diluted tenfold in PBS and aliquots were plated onto LB agar. The number of intracellular bacteria was determined as CFU.

Terminal Ileo biopsies were obtained and treated as described above. After dissociation cells were resuspended at a density of 1E+06/mL in RPMI supplemented with 10% FBS, 2 mM l-glutamine, 0.1 mM non-essential amino acid (Sigma) and 30 μM iron (III) citrate or 30 μM DFO were required and seeded 250 µL/well in a 96-well plate round bottom. The following day, cells were washed and resuspended in antibiotic-free medium supplemented with iron (III) citrate or DFO. Cells were infected with STM-LT2, STM-D23580 or ST-Ty2 at a MOI of 5 for 2 h, then 100 μg/mL gentamicin was added for 30 min. At 2.5 h p.i. supernatants were collected and stored at −80 °C. Cells were then washed and resuspended in a fresh medium containing 30 μg/mL gentamicin for the remaining of the experiments. The number of intracellular bacteria was determined at 4 h p.i.

### ELISAs

IL-12p70, IL-1β and INF-γ levels were measured from culture supernatants by ELISA kits (R&D System) according to the manufacturer’s instructions. The range for IL-12p70 was 2000–31.2 pg/mL, for IL-1β was 250–1.9 pg/mL and for INF-γ was 1000–15.6 pg/mL. The results were expressed as pg/mL for each cytokine.

### ROI staining

MoDCs were pre-treated with iron (III) citrate or DFO and infected with STM-LT2, STM-D23580 or ST-Ty2 as described above. At 6 h p.i., cells were washed and resuspended in serum-free medium supplemented with 5 μM of CellROX^®^ Green reagent (ThermoFisher) and incubated at 37 °C for 30 min. Cells were then washed and incubated for 30 min with anti-CD71 at a concentration of 1:100 (PeCy7; CY1G4, BioLegend). The LIVE/DEAD™ Fixable Near-IR Dead Cell Stain Kit (ThermoFisher) was used for the exclusion of dead cells. After incubation, cells were washed in FACS buffer and fixed in 3.7% formaldehyde. Samples were acquired on a Fortessa X20 flow cytometer (BD Biosciences) and files were analysed on Flowjo (v.10.4.1).

### Hydrogen peroxide and Spermin NONOate stimulation assay

Bacteria were grown overnight in LB broth and diluted 1:33 in PBS. Bacteria were stimulated with 0.1 mM H_2_O_2_ (Sigma) 0.5 mM Spermin NONOate (Sigma) or left unstimulated for 5 min, 15 min or 60 min. At each time, tenfold dilutions of the bacterial suspension were plated, in quadruplicate, on LB Lennox agar (Sigma). The number of bacteria was determined as Colony-forming Units (CFU).

### Siderophore estimation assay

Siderophore production was assessed by chrome azurol sulphonate (CAS) assay. Before starting, glassware was rinsed with 3 mol/l hydrochloric acid (HCl) to remove iron and subsequently washed in deionized water. CAS reagent was prepared as described in^88^. Briefly, 121 mg CAS (Sigma) was dissolved in 100 ml distilled water and 20 mL of 1 mM ferric chloride (MP) solution prepared in 10 mM HCl. This solution was added to 20 ml hexadecyl trimethyl ammonium bromide (HDTMA, Acros Organics) solution under stirring. HDTMA solution was prepared by mixing 182.25 mg HDTMA in 100 ml distilled water. The CAS-HDTMA solution was sterilised before further use. Bacteria were grown overnight in LB broth and diluted 1:33 in fresh LB broth or Davis-Minimum Medium (Sigma) supplemented with 0.1 mM H_2_O_2_ or 0.25 mM Spermin NONOate and culture supernatants were collected at each time point. In total, 100 µL of supernatants were mixed with 100 μL of CAS reagent in a 96-well plate and the optical density of each sample was recorded at 630 nm using CLARIOStar spectrophotometer. Three replicates were taken for each strain.

Siderophore production was measured in percent siderophore unit (psu) which was calculated according to the following formula^89^: [(A_R_ − A_S_) × 100]/A_R_, where A_R_ = absorbance of reference (CAS solution and uninoculated broth), and A_S_ = absorbance of the sample (CAS solution and cell-free supernatant of sample).

### Statistics and reproducibility

Statistical analyses were performed using GraphPad-Prism7 (GraphPad Software, San Diego, CA, USA). Differences among groups were determined by one-way or two-way ANOVA, as appropriate. ANOVA test statistics were corrected post hoc by Tukey, applying a 95% confidence interval. A *P* value <0.05 was considered statistically significant. All other analyses were performed in the *R* statistical programming environment, using the latest version of *R* and *Bioconductor* packages as appropriate. The number of biological samples used for each experiment is mentioned in the respective figure legends.

### Reporting summary

Further information on research design is available in the Nature Research Reporting Summary linked to this article.

## Supplementary information

Supplementary Information

Description of Additional Supplementary Files

Supplementary Data 1

Supplementary Data 2

Supplementary Data 3

Supplementary Data 4

Supplementary Data 5

Supplementary Data 6

Supplementary Data 7

Supplementary Data 8

Supplementary Data 9

Supplementary Data 10

Reporting Summary

## Data Availability

Sequences data have been deposited in the Gene Expression Omnibus under GSE161854. All experimental data are available from the authors. The source data underlying the graphs and charts in the figure are shown in Supplementary Data 8–10.
