# Supplementary material for: Dual RNA sequencing reveals dendritic cell reprogramming in response to typhoidal Salmonella invasion
Source: Commun Biol. 2022 Feb 4;5:111. doi: 10.1038/s42003-022-03038-z (PMC8816929; doi:10.1038/s42003-022-03038-z)
Supplement: Supplementary file 1 — Supplementary Information [file 42003_2022_3038_MOESM1_ESM.pdf]

Supplementary figures for:

Dual RNA sequencing reveals dendritic cell reprogramming in response to typhoidal *Salmonella* invasion

Anna Aulicino[1,2,§] & Agne Antanaviciute[1,2,3,§], Joe Frost[1], Ana Sousa-Geros[1,2], Esther Mellado[4], Moustafa Attar[4,5], Marta Jagielowicz[1,2], Philip Hublitz[6], Julia Sinz[1,2], Lorena Preciado-Llanes[1,2], Giorgio Napolitani[1], Rory Bowden[4], Hashem Koohy[1,3], Hal Drakesmith[1] and Alison Simmons[1,2,*].

[1] *MRC Human Immunology Unit, MRC Weatherall Institute of Molecular Medicine, University of Oxford, Oxford OX3 9DS, UK*

[2] *Translational Gastroenterology Unit, John Radcliffe Hospital, Headington, Oxford OX3 9DU, UK*

[3] *MRC WIMM centre for Computational Biology, Weatherall Institute of Molecular Medicine, University of Oxford, Oxford OX3 9DS, UK,*

[4] *Wellcome Centre for Human Genetics, University of Oxford, Roosevelt Drive, Headington, Oxford, OX3 7BN, UK*

[5] *Kennedy Institute of Rheumatology, University of Oxford, Roosevelt Drive, Headington, Oxford, OX3 7FY, UK*

[6] *MRC Weatherall Institute of Molecular Medicine, Genome Engineering Facility, University of Oxford, Oxford OX3 9DS, UK*

[*] *Corresponding author:* alison.simmons@ndm.ox.ac.uk

[§] *These authors contributed equally to this work.*

Supplementary Figure 1

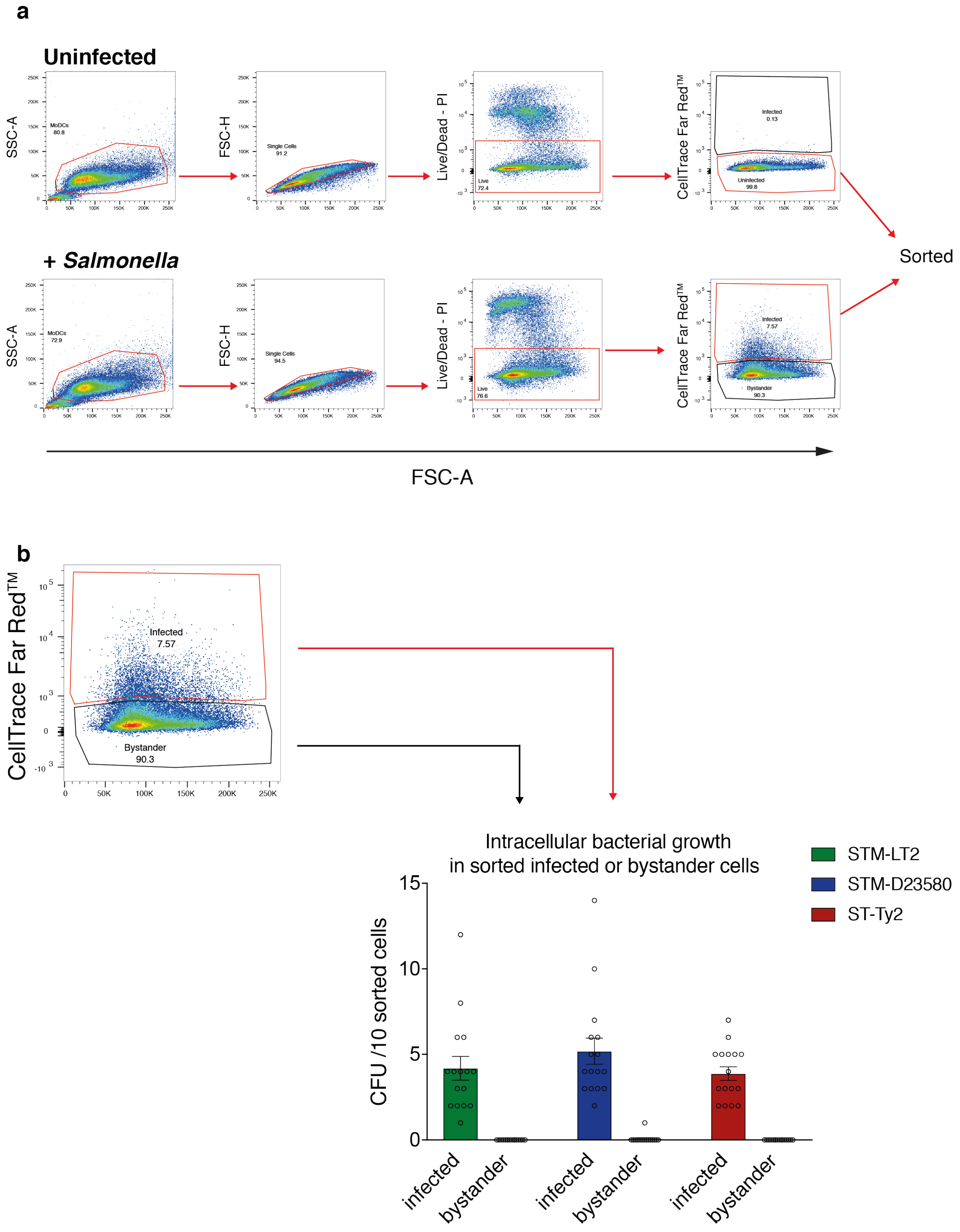

Supplementary Figure 1. FACS plots of uninfected and *Salmonella*-challenged MoDCs.

a Representative example of the gating strategy used to sort uninfected or infected MoDCs. The upper gate displaying a high CellTrace™ FarRed fluorescence intensity captures MoDCs that engulfed *Salmonella* (Infected), while the lower gate (Bystander) includes the MoDCs that did not exhibit fluorescence. **b** For each experiment, 10 single cells per each experimental condition were sorted in 8-well strips containing 1% saponin in PBS and plated onto agar plates to determine the number of intracellular CFU. The mean ± SEM from sixteen independent experiments is shown. Two-way ANOVA test.

Supplementary Figure 2

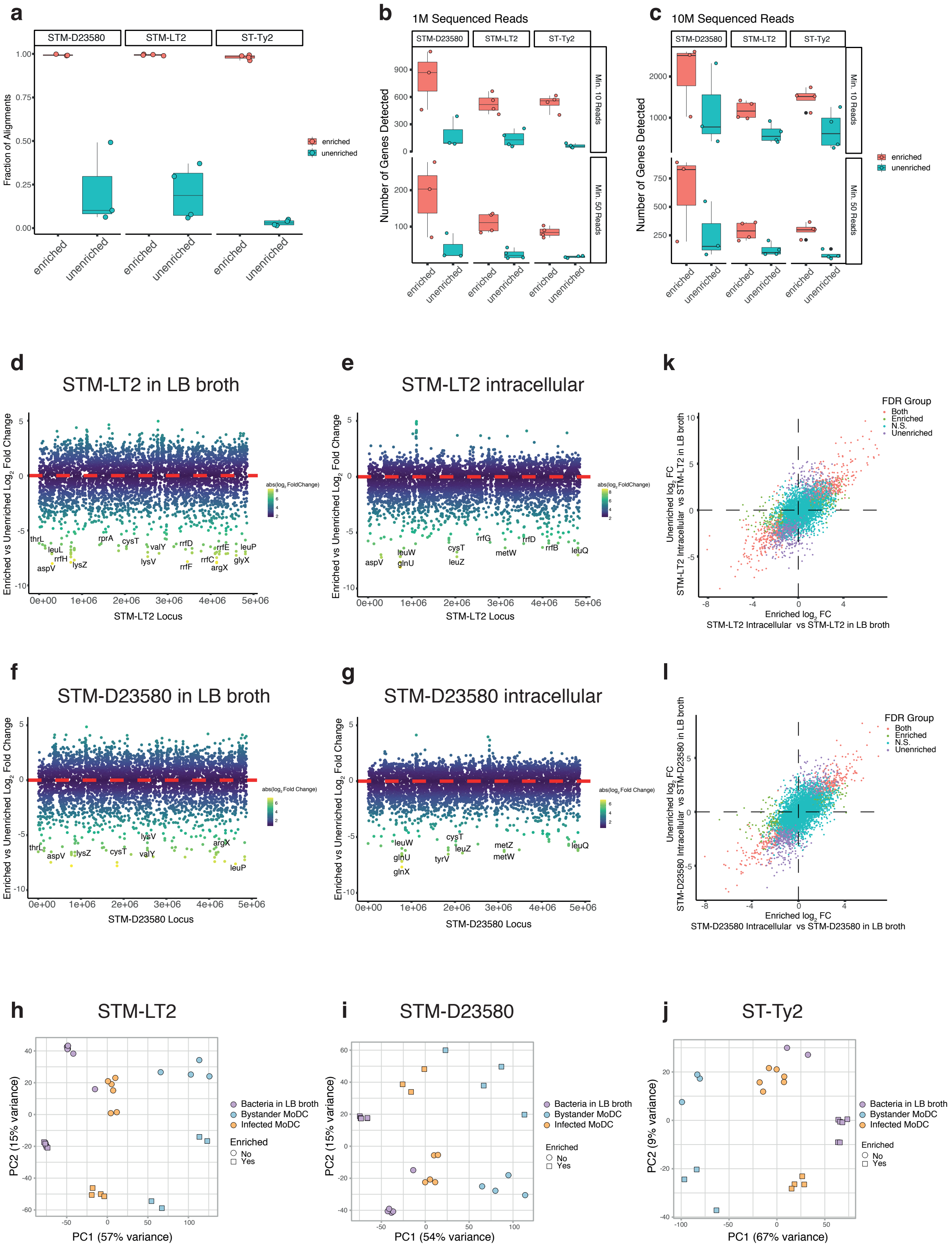

Supplementary Figure 2. Quality control

a Boxplots show fraction of all reads aligned to joint *Salmonella*-human reference mapping to bacterial chromosome of STM-D23580, STM-LT2 and ST-Ty2 respectively using enrichment strategy vs unenriched libraries. Individual points are shown over boxplot.

b-c Sequencing economy comparison of bacterial gene recovery above coverage thresholds required for robust comparisons. Box plots show the number of bacterial genes detected above 10 and 50 minimum read thresholds using enrichment strategy vs unenriched libraries in STM-D23580, STM-LT2 and ST-Ty2. All libraries were down sampled equally to 1 million starting reads (**b**) or 10 million starting reads (**c**). Median, 25th and 75th percentiles are shown.

Scatter plot of log2 fold change values of all bacterial genes between enriched vs unenriched library strategies applied to STM-LT2 grown in LB broth (**d**) and intracellular bacteria (**e**) and STM-D23580 grown in LB broth (**f**) and intracellular bacteria (**g**). Genes are plotted according to their chromosome locus, with no major locus-bias observed in the enrichment probes. Selected ribosomal and transfer RNAs, which were excluded from the probe design in order to deplete these unwanted species, are labelled and show > 32 fold depletion in enrichment libraries.

Preservation of biological signal using enrichment strategy. Principal component analysis using bacterial gene expression of all samples containing or exposed to *Salmonella* STM-LT2 (**h**), STM-D23580 (**i**) or ST-Ty2 (**j**). The first principal component, which account for the majority of the variance in the data, separates three biological groups (bacteria grown in LB broth, infected MoDCs or bystander MoDCs) in all three strains, while the differences between enriched and unenriched library strategies are reflected in the second principal component (between 9-15% of total variance). As enriched and unenriched libraries were sequenced separately, sequencing batch effect is also encompassed by the second PC.

Preservation of biological signal using enrichment strategy. Scatter plot shows the correlation of intra vs extra cellular comparison log_2 fold changes between enriched and non-enriched library strategies in STM-LT2 (**k**) and STM-D23580 (**l**). Genes are grouped as either found to be non-differentially expressed in either library strategy (N.S), differentially expressed by both library strategies (Both) or

only differentially expressed in one library strategy (Enriched/Unenriched). In most cases, the direction of the fold-changes correlates, even if significance can only be attributed only in one library strategy. Most cases where the direction of fold-changes is opposed between library strategies fall into N.S category, which contains the majority of low expression, high-variance genes.

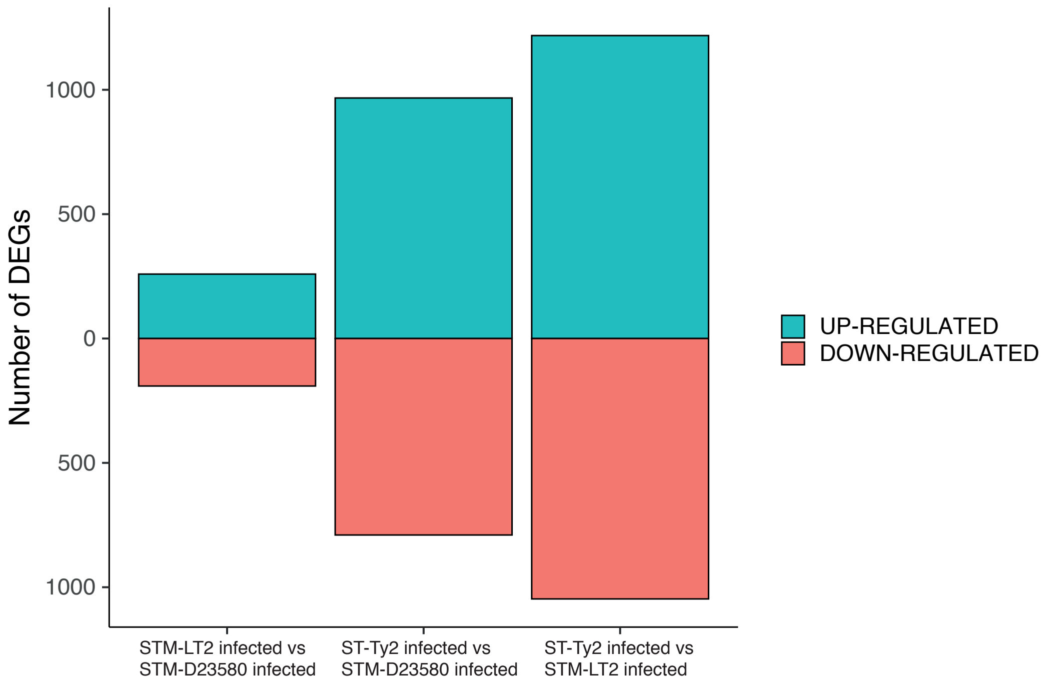

Supplementary Figure 3. Differentially Expressed genes identified between STM-D23580, STM-LT2 and ST-Ty2 infected MoDCs

Histogram showing the up- and down- regulated gene numbers in each of the three pairwise comparison. Count of genes significantly up- or down- regulated in the infected MoDCs are indicated above and below the x-axis, respectively.

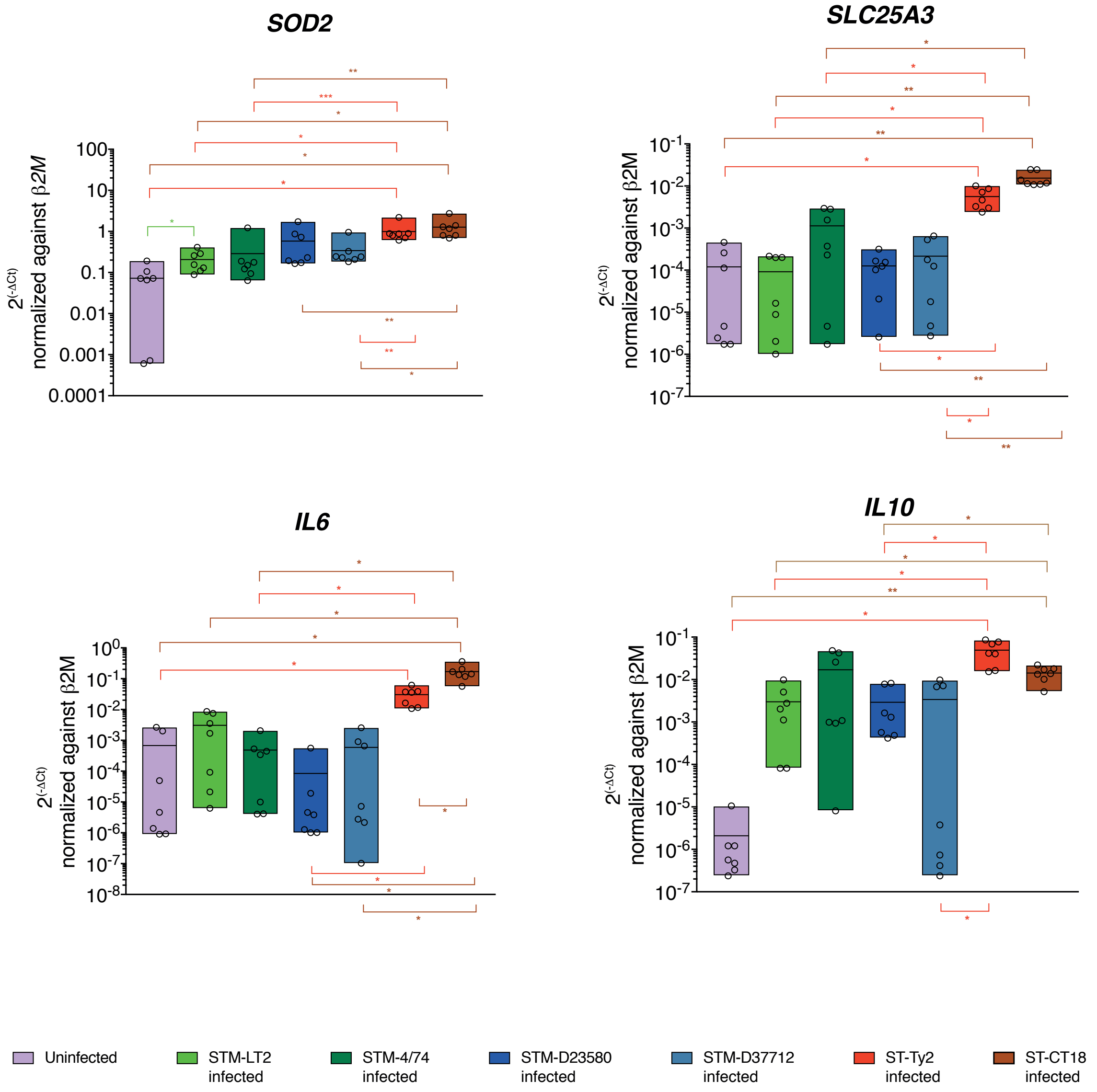

Supplementary Figure 4. qPCR data validate RNA-seq findings in multiple bacterial strains

Gene expression of *SOD2, SLC25A3, IL6 and IL10* measured by qPCR at 6h p.i. in MoDCs infected with multiple *Salmonella* strains or left uninfected. Mean ± SEM from seven independent experiments are shown. Two-way ANOVA test, *P*-value < 0.05 (*), < 0.01 (**), < 0.001 (***).

Supplementary Figure 5

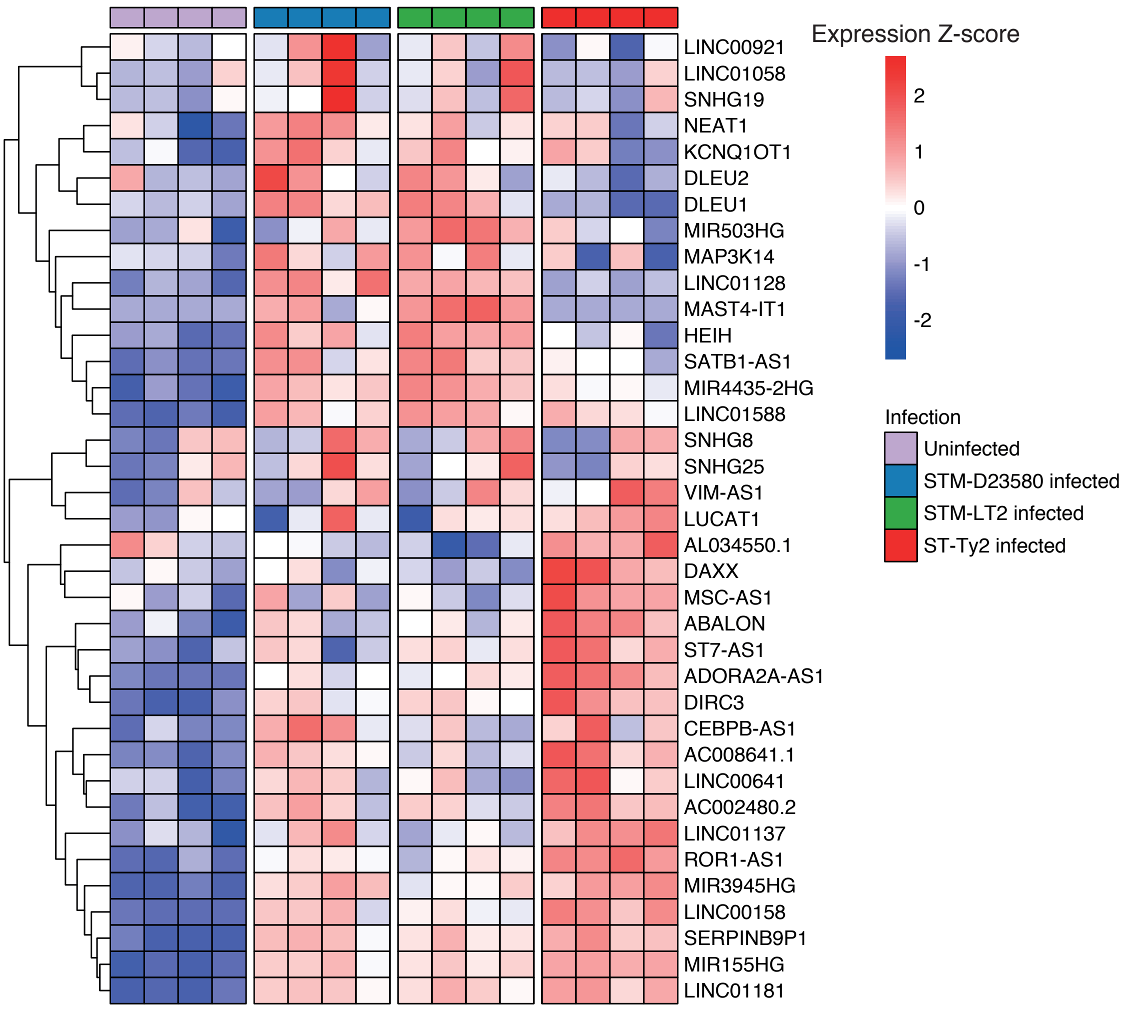

Supplementary Figure 5. Differential expression of lncRNA

Heat map showing changes in the expression of lncRNA in MoDCs upon *Salmonella* infection compared to uninfected cells. Scaled, variance-stabilised, log-transformed data are shown, with expression colour coded according to the key provided on the right.

Supplementary Figure 6

a

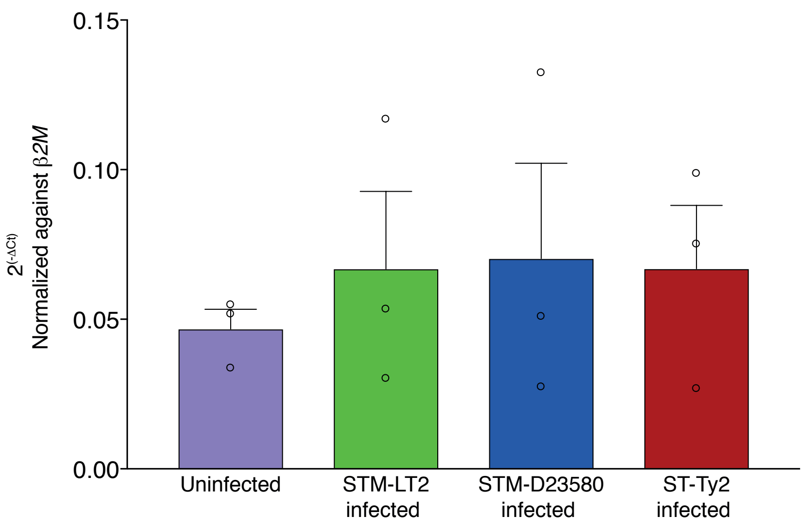

b

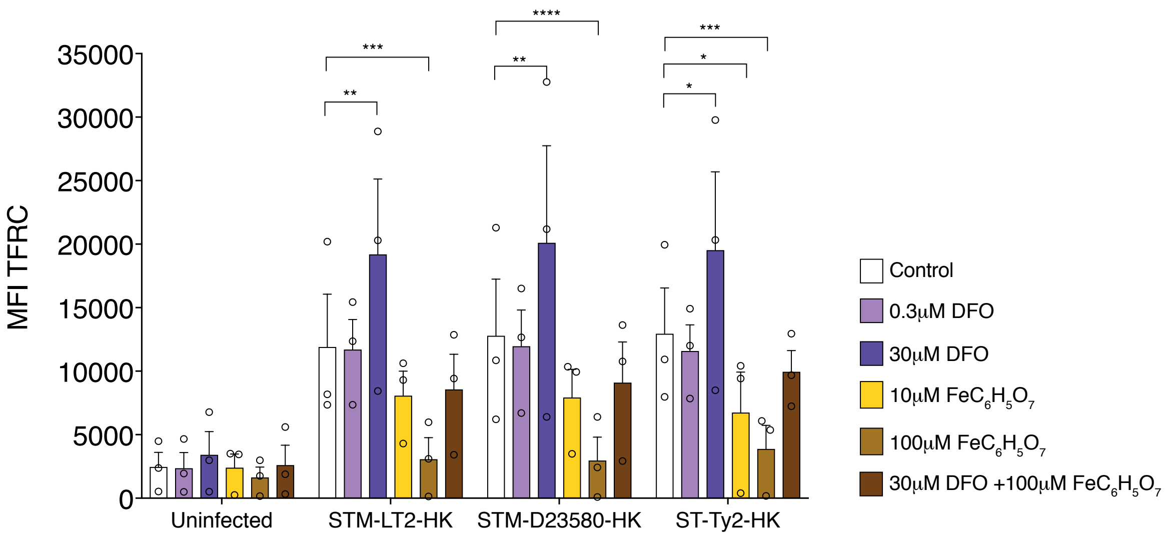

Supplementary Figure 6. *TFRC* gene expression and its modulation by DFO and $FeC_6H_5O_7$

a Gene expression of *TFRC* measured by qPCR at 24h p.i. in MoDCs infected with STM-LT2, STM-D23580 or ST-Ty2 or left uninfected. Mean ± SEM from three independent experiments are shown.

b MoDCs pre-treated with $FeC_6H_5O_7$ or the iron chelator DFO were activated by stimulation with heat-killed (HK) bacteria for 24h and the surface expression of TFRC was measured by flow cytometry. The mean ± SEM from three independent experiments is shown. Two-way ANOVA test, *P*-value < 0.05 (*), < 0.01 (**), < 0.001 (***).

Supplementary Figure 7

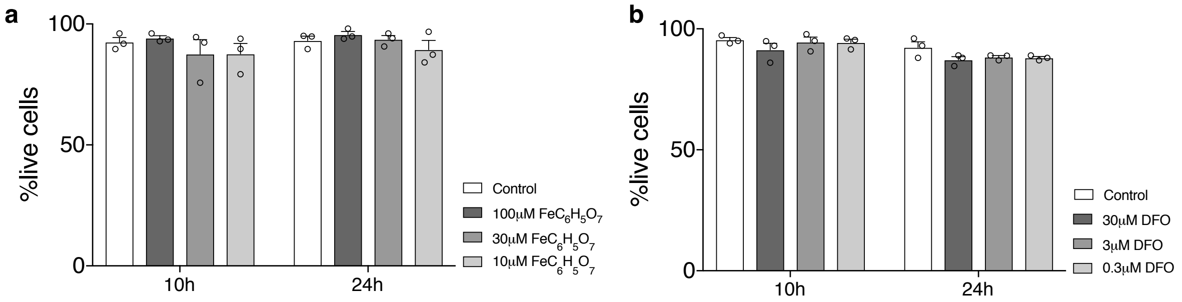

Supplementary Figure 7. MoDCs viability is not affected by DFO or $FeC_6H_5O_7$ pre-treatment

MoDCs were stimulated with $FeC_6H_5O_7$ (**a**) or the iron chelator DFO (**b**) for 24h and cell viability was assessed by trypan blue exclusion. The mean ± SEM from three independent experiments is shown. Two-way ANOVA test.

Supplementary Figure 8

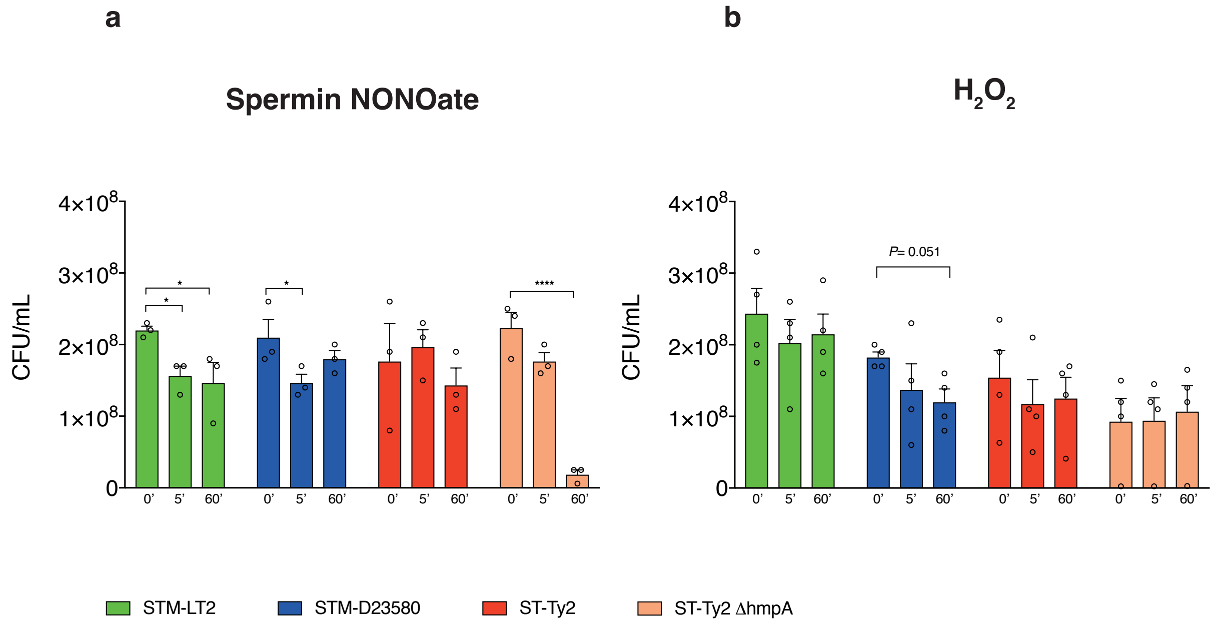

Supplementary Figure 8. ST-Ty2 ΔhmpA is susceptible to nitrosative stress

Salmonella surviving after challenge with 0.5mM Spermin NONOate (**a**) or 0.1mM hydrogen peroxide (H_2O_2) (**b**) for 5 or 60min. The mean ± SEM from three and four independent experiments is shown. Two-way ANOVA test, P-value < 0.05 (*), < 0.0001 (****).

Supplementary Figure 9

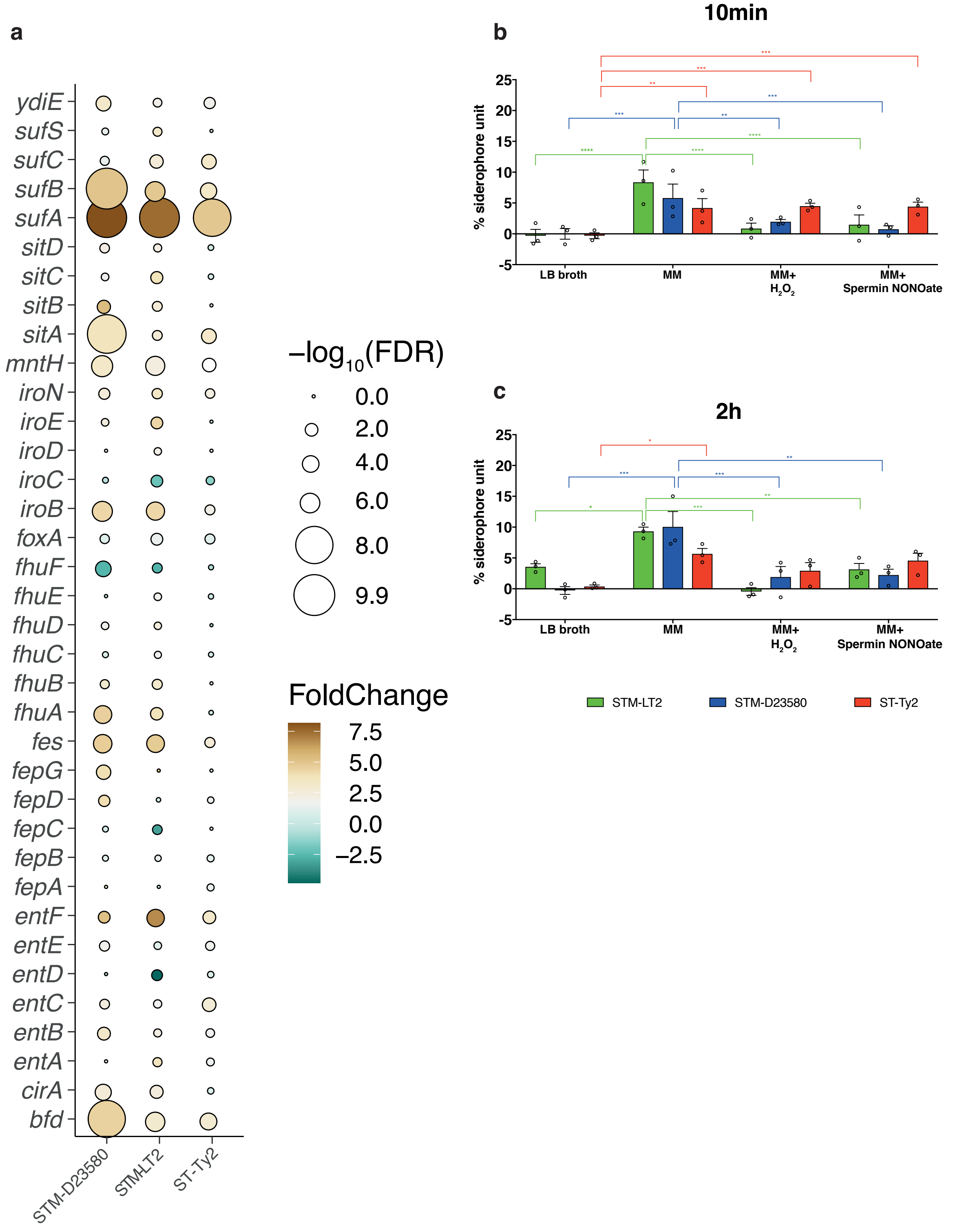

Supplementary Figure 9. Expression of Iron acquisition systems

a Dotplot heatmap showing iron-related gene expression in intracellular *Salmonella* relative to bacteria grown in LB broth. Points are sized by the significance of the observation ($-\log_{10}$ FDR) and coloured by the average $\log_2$ fold change.

Bacterial siderophore production over 10min (**b**) or 2h (**c**) growth in LB broth or minimal medium (MM) supplemented with 0.1mM of H_2O_2 or 0.5mM of Spermin NONOate. Mean ± SEM from three independent experiments are shown. Two-way ANOVA test, *P*-value < 0.05 (*), < 0.01 (**), < 0.001 (***), < 0.0001 (****).

Supplementary Figure 10

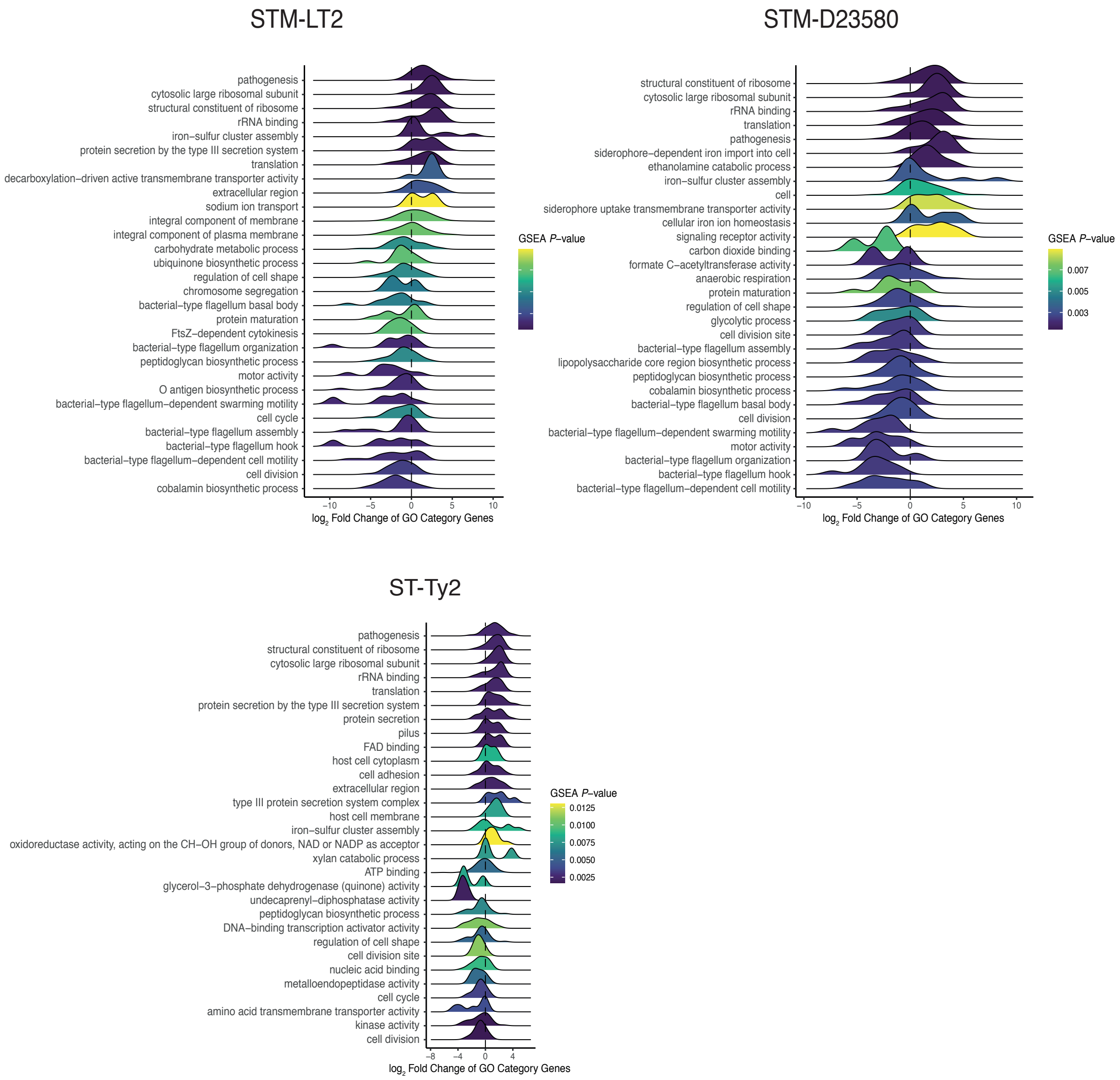

Supplementary Figure 10. Enriched categories by intracellular *Salmonella*

Ridge plots showing the top 20 significantly altered pathways in the *Salmonella* strains during infection, ordered by $\log_2$ fold changes of genes within each category and coloured by GSEA *P*-value.

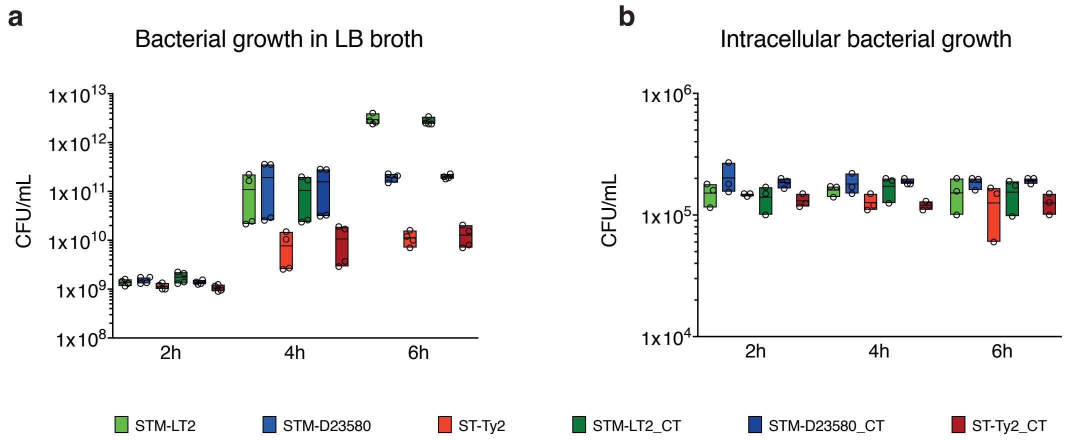

Supplementary Figure 11. CellTrace™ Far Red proliferation kit does not affect bacterial viability and invasion

a Bacteria were stained with CellTrace™ Far Red (CT) and grown in LB broth in comparison to unstained bacteria. Mean ± SEM from four independent experiments are shown. Two-way ANOVA test.

b MoDCs were infected with stained or unstained bacteria and CFU were assessed at 2h, 4h and 6h p.i. Mean ± SEM from three independent experiments are shown. Two-way ANOVA test.
