# Supplementary material for: Dual RNA sequencing reveals dendritic cell reprogramming in response to typhoidal Salmonella invasion
Source: Commun Biol. 2022 Feb 4;5:111. doi: 10.1038/s42003-022-03038-z (PMC8816929; doi:10.1038/s42003-022-03038-z)
Supplement: Supplementary file 2 — Description of Additional Supplementary Files [file 42003_2022_3038_MOESM2_ESM.pdf]

Description of Additional Supplementary Files

File name: Supplementary Data 1

Description: Bacterial probe sequence

File name: Supplementary Data 2

Description: Bacterial DE genes

File name: Supplementary Data 3

Description: DE genes between infected and uninfected MoDCs

File name: Supplementary Data 4

Description: Host_infected_vs_Uninfected GO

File name: Supplementary Data 5

Description: DEG_comparison_Infected MoDC

File name: Supplementary Data 6

Description: lncRNA

File name: Supplementary Data 7

Description: Bacterial GSEA

File name: Supplementary Data 8

Description: Source Data Figure 4

File name: Supplementary Data 9

Description: Source Data Figure 5

File name: Supplementary Data 10

Description: Source Data Figure 6
