# Supplementary material for: Dual RNA sequencing reveals dendritic cell reprogramming in response to typhoidal Salmonella invasion
Source: Commun Biol. 2022 Feb 4;5:111. doi: 10.1038/s42003-022-03038-z (PMC8816929; doi:10.1038/s42003-022-03038-z)
Supplement: Supplementary file 13 — Reporting Summary [file 42003_2022_3038_MOESM13_ESM.pdf]

nature research

Corresponding author(s): Prof. Alison Simmons

Last updated by author(s): Nov 15, 2021

Reporting Summary

Nature Research wishes to improve the reproducibility of the work that we publish. This form provides structure for consistency and transparency in reporting. For further information on Nature Research policies, see our Editorial Policies and the Editorial Policy Checklist.

Statistics

For all statistical analyses, confirm that the following items are present in the figure legend, table legend, main text, or Methods section.

n/a	Confirmed	
☐	☒	The exact sample size (*n*) for each experimental group/condition, given as a discrete number and unit of measurement
☒	☐	A statement on whether measurements were taken from distinct samples or whether the same sample was measured repeatedly
☐	☒	The statistical test(s) used AND whether they are one- or two-sided<br>*Only common tests should be described solely by name; describe more complex techniques in the Methods section.*
☐	☒	A description of all covariates tested
☐	☒	A description of any assumptions or corrections, such as tests of normality and adjustment for multiple comparisons
☐	☒	A full description of the statistical parameters including central tendency (e.g. means) or other basic estimates (e.g. regression coefficient) AND variation (e.g. standard deviation) or associated estimates of uncertainty (e.g. confidence intervals)
☐	☒	For null hypothesis testing, the test statistic (e.g. *F*, *t*, *r*) with confidence intervals, effect sizes, degrees of freedom and *P* value noted<br>*Give P values as exact values whenever suitable.*
☒	☐	For Bayesian analysis, information on the choice of priors and Markov chain Monte Carlo settings
☐	☒	For hierarchical and complex designs, identification of the appropriate level for tests and full reporting of outcomes
☐	☒	Estimates of effect sizes (e.g. Cohen's *d*, Pearson's *r*), indicating how they were calculated

Our web collection on statistics for biologists contains articles on many of the points above.

Software and code

Policy information about availability of computer code

Data collection	No software was used to collect data in this study.
Data analysis	The following softwares and R packages were used fo data analysis:<br>mauve software (version 2.4.0), fastQC software (version 0.11.9), cutadapt software (version1.16), STAR aligner (version 2.4.2a), featureCounts (version 1.6.2), SeqTK (version 1.0-r68e), DESeq2 R package (version 1.24), clusterProfiler (version 3.12.0).<br>GraphPad Prism 7 was used statistical analyses. Flowjo (version 10.4.1) was used for analysis of flow cytometry data.

For manuscripts utilizing custom algorithms or software that are central to the research but not yet described in published literature, software must be made available to editors and reviewers. We strongly encourage code deposition in a community repository (e.g. GitHub). See the Nature Research guidelines for submitting code & software for further information.

Data

Policy information about availability of data

All manuscripts must include a data availability statement. This statement should provide the following information, where applicable:

- Accession codes, unique identifiers, or web links for publicly available datasets
- A list of figures that have associated raw data
- A description of any restrictions on data availability

Sequences data has been deposited in the Gene Expression Omnibus under GSE161854. All experimental data are available from the authors. Request for materials and data should be addressed to A.S.

Field-specific reporting

Please select the one below that is the best fit for your research. If you are not sure, read the appropriate sections before making your selection.

- [x] Life sciences
- [ ] Behavioural & social sciences
- [ ] Ecological, evolutionary & environmental sciences

For a reference copy of the document with all sections, see nature.com/documents/nr-reporting-summary-flat.pdf

Life sciences study design

All studies must disclose on these points even when the disclosure is negative.

Sample size	No statistical methods were used to determine sample size.<br>48 bulk samples were generated from 6 independent donors.
Data exclusions	Outliers were detected by PCA as described in methods, and were found to significantly correlate with poor quality libraries by multiple QC metrics. As such, two were excluded from host transcriptome differential expression analysis.
Replication	Reported results were consistently replicated across multiple experiments with all replicates generating similar results.<br>Figure 3a and 3b: Four independent experiments are shown.<br>Figure 4a: Four independent experiments are shown.<br>Figure 4b, 4c, 4d: Three independent experiments are shown.<br>Figure 4e, 4f: Six independent experiments are shown.<br>Figure 5a: Four independent experiments are shown.<br>Figure 5c: Three independent experiments are shown.<br>Figure 5d: Five independent experiments are shown.<br>Figure 5f: Four independent experiments are shown.<br>Figure 5g: Five independent experiments are shown.<br>Figure 6a: Four independent experiments are shown.<br>Figure 6b: Five independent experiments are shown.<br>Figure 6c: Three independent experiments are shown.
Randomization	RNA-seq: to avoid lane effects the distribution of the samples was randomized on the sequencing platform
Blinding	Investigators were not blinded.

Reporting for specific materials, systems and methods

We require information from authors about some types of materials, experimental systems and methods used in many studies. Here, indicate whether each material, system or method listed is relevant to your study. If you are not sure if a list item applies to your research, read the appropriate section before selecting a response.

Materials & experimental systems

n/a	Involved in the study	
[ ]	[x]	Antibodies
[x]	[ ]	Eukaryotic cell lines
[x]	[ ]	Palaeontology and archaeology
[x]	[ ]	Animals and other organisms
[ ]	[x]	Human research participants
[x]	[ ]	Clinical data
[x]	[ ]	Dual use research of concern

Methods

n/a	Involved in the study	
[x]	[ ]	ChIP-seq
[ ]	[x]	Flow cytometry
[x]	[ ]	MRI-based neuroimaging

Antibodies

Antibodies used	anti-CD71 (PeCy7; CY1G4, BioLegend)
Validation	Antibodies were validated by the suppliers.

Human research participants

Policy information about studies involving human research participants

Population characteristics	Leukocyte Reduction System cones were obtained from healthy donors from the UK National Blood Centre.
Recruitment	Leukocyte Reduction System cones were obtained from healthy donors from the UK National Blood Centre.
Ethics oversight	All protocols for recruitment of human subjects and use of human terminal ileum biopsies were approved by NHS National Research Ethics Service (NRES) research ethics committee (REC) references for the study include 18/WM/0237. Protocol number: 13463. IRAS project ID: 243653.

Note that full information on the approval of the study protocol must also be provided in the manuscript.

Flow Cytometry

Plots

Confirm that:

- [x] The axis labels state the marker and fluorochrome used (e.g. CD4-FITC).
- [x] The axis scales are clearly visible. Include numbers along axes only for bottom left plot of group (a 'group' is an analysis of identical markers).
- [x] All plots are contour plots with outliers or pseudocolor plots.
- [x] A numerical value for number of cells or percentage (with statistics) is provided.

Methodology

Sample preparation	Cells were washed in FACS buffer containing PBS, 0.3% (v/v) Bovine Serum Albumin (BSA, Sigma) and 2mM EDTA (Invitrogen) and incubated for 30min with the Antibodies of interest.
Instrument	Fortessa X20 flow cytometer (BD Biosciences)
Software	Flowjo (v.10.4.1)
Cell population abundance	Infected MoDCs represented 10% while bystander MoDCs represented 90% of the live cells.
Gating strategy	MoDCs were fisrt identified in a forward scatter (FSC) and side scatter (SSC) plot. The doublets and the dead cells were excluded. Next, the infected and bystander cells were identified based on the fluorescence of the FarRed dye.

- [x] Tick this box to confirm that a figure exemplifying the gating strategy is provided in the Supplementary Information.
